# Structural Evolution of Small-Sized Phosphorus-Doped Boron Clusters: A Half-Sandwich-Structured PB_15_ Cluster

**DOI:** 10.3390/molecules29143384

**Published:** 2024-07-18

**Authors:** Danyu Wang, Yueju Yang, Shixiong Li, Deliang Chen

**Affiliations:** School of Physics and Electronic Science, Guizhou Education University, Guiyang 550018, China; 18508507005@163.com (D.W.); yangyueju1003@163.com (Y.Y.); chendeliang@gznc.edu.cn (D.C.)

**Keywords:** boron clusters, phosphorus-doped, geometrical structures, spectra

## Abstract

The present study is a theoretical investigation into the structural evolution, electronic properties, and photoelectron spectra of phosphorus-doped boron clusters PB_n_^0/−^ (n = 3–17). The results of this study revealed that the lowest energy structures of PB_n_^−^ (n = 3–17) clusters, except for PB_17_^−^, exhibit planar or quasi-planar structures. The lowest energy structures of PB_n_ (n = 3–17), with the exceptions of PB_7_, PB_9_, and PB_15_, are planar or quasi-planar. The ground state of PB_7_ has an umbrella-shaped structure, with C_6V_ symmetry. Interestingly, the neutral cluster PB_15_ has a half-sandwich-like structure, in which the P atom is attached to three B atoms at one end of the sandwich, exhibiting excellent relative and chemical stability due to its higher second-order energy difference and larger HOMO–LUMO energy gap of 4.31 eV. Subsequently, adaptive natural density partitioning (AdNDP) and electron localization function (ELF) analyses demonstrate the bonding characteristics of PB_7_ and PB_15_, providing support for the validity of their stability. The calculated photoelectron spectra show distinct characteristic peaks of PB_n_^−^ (n = 3–17) clusters, thus providing theoretical evidence for the future identification of doped boron clusters. In summary, our work has significant implications for understanding the structural evolution of doped boron clusters PB_n_^0/−^ (n = 3–17), motivating further experiments regarding doped boron clusters.

## 1. Introduction

It is well known that boron is a naturally abundant element, and scientists have been exploring the properties and applications of boron, boron compounds, and their derivatives [1,2,3,4,5]. With the successive discoveries of fullerene C_60_, carbon nanotubes, and graphene [6,7,8], carbon nanomaterials have attracted considerable attention and become a dynamic field of research; similarly, as a neighbor of carbon, boron has also received significant attention from researchers. Boron compounds exhibit intrinsic electronic defects, as well as the ability to form an outstanding number of multicenter bonds, which endow them with rich structural features and chemical properties [9,10,11,12,13,14]. The groundbreaking discovery of borospherene [15] in 2014 sparked extensive research on boron clusters [16,17,18,19,20,21,22]. Over the past two decades, a great deal of theoretical and experimental research has focused on the geometric structure and electronic characteristics of bare boron clusters [23]. It is generally believed that bare boron clusters exhibit planar or quasi-planar geometric shapes over a large range of sizes. One of the most important discoveries is the planar B_36_^−^ cluster [24], a highly stable cluster with central hexagonal vacancies that can be synthesized on a metal substrate to form “borophene”. The experimental discoveries of borospherene and borophene provide new insights into novel boron nanomaterials and nanodevices.

Over the past decade, scientists have investigated richly doped boron clusters, with a primary focus on doping single metal atoms into boron clusters of various sizes. Metal doping is an effective method for adjusting the chemical bonds and occupied energy levels of boron clusters through the addition of metal elements, thereby changing their physicochemical properties. Doping metal atoms into boron clusters can lead to the formation of new geometric configurations and chemical properties, such as ring-like, sandwich-like, tube-like, and cage-like structures [17,19,20,25,26,27,28,29,30,31,32,33,34]. Following the doping of single alkali metal atoms into the quasi-planar structures of B_20_^−^ and B_22_^−^ [35], species including LiB_20_^−^, NaB_22_^−^, and KB_22_^−^ exhibit bi-ring structures [33,36]. Quasi-planar B_12_^−^ clusters, upon doping with single transition metal atoms of Co, Rh, or Ta, exhibit half-sandwich structures [10,37,38]. Doping with single transition metal atoms can modify the bi-ring tubular B_24_ into cage-like boron clusters (TiB_24_, VB_24_, and MnB_24_) and tri-ring tubular doped boron clusters (ScB_24_) [39,40,41], or convert quasi-planar B_24_^−^ into cage-like boron clusters (TiB_24_^−^, CrB_24_^−^, and VB_24_^−^) [35,42].

Compared with metal-doped boron clusters, there is relatively little research on the doping of boron clusters with non-metal atoms [43,44,45,46,47]. In particular, there have been few studies on the structural evolution of boron clusters with the addition of phosphorus atoms. The recently reported P-doped boron cluster P_2_B_12_^+/0/−^ has the same cage structure, with D_3h_ symmetry [46]. The theoretical study of phosphorus-doped small boron clusters is of great significance for the discovery of new structures and properties of boron clusters. In this study, the effect of P-atom doping on the structure and electronic properties of boron clusters B_n_^0/−^ (n = 3–17) was investigated through employment of the particle swarm optimization (CALYPSO) method [48] and the density-functional theory method PBE0 [49].

## 2. Results and Discussion

### 2.1. Geometric Configurations

**Figure 1:**
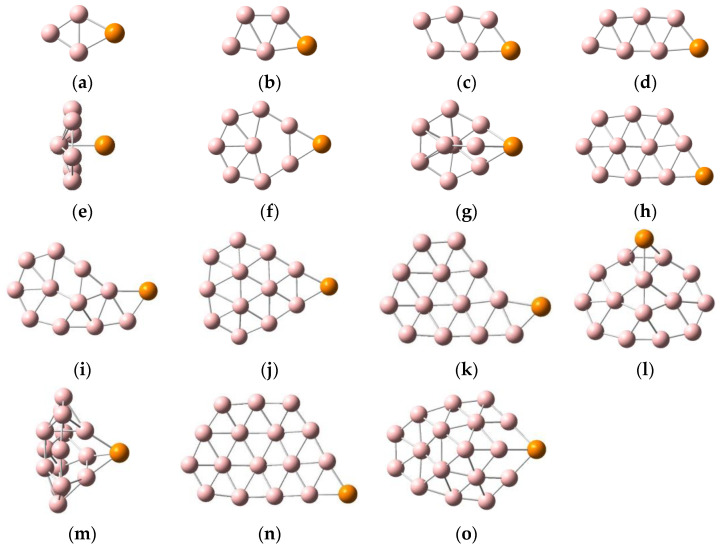
Structures of PB_n_, where pink balls represent boron atoms and orange ball represents phosphorus atom. (**a**) PB_3_ C_2V_; (**b**) PB_4_ C_S_; (**c**) PB_5_ C_S_; (**d**) PB_6_ C_S_; (**e**) PB_7_ C_6V_; (**f**) PB_8_ C_2V_; (**g**) PB_9_ C_S_; (**h**) PB_10_ C_1_; (**i**) PB_11_ C_1_; (**j**) PB_12_ C_S_; (**k**) PB_13_ C_1_; (**l**) PB_14_ C_S_; (**m**) PB_15_ C_S_; (**n**) PB_16_ C_1_; (**o**) PB_17_ C_S_.

**Figure 2:**
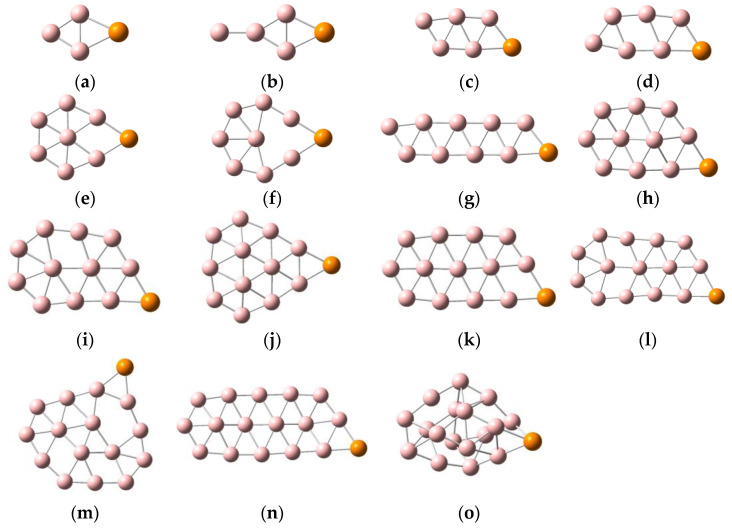
Structures of PB_n_^−^, where pink balls represent boron atoms and orange ball represents phosphorus atom. (**a**) PB_3_^−^ C_2V_; (**b**) PB_4_^−^ C_2V_; (**c**) PB_5_^−^ C_S_; (**d**) PB_6_^−^ C_S_; (**e**) PB_7_^−^ C_2V_; (**f**) PB_8_^−^ C_2V_; (**g**) PB_9_^−^ C_S_; (**h**) PB_10_^−^ C_1_; (**i**) PB_11_^−^ C_S_; (**j**) PB_12_^−^ C_S_; (**k**) PB_13_^−^ C_1_; (**l**) PB_14_^−^ C_S_; (**m**) PB_15_^−^ C_1_; (**n**) PB_16_^−^ C_1_; (**o**) PB_17_^−^ C_1_.

Early theoretical and experimental studies found that anionic boron clusters with an atomic numbers less than 37 always maintain quasi-planar or planar structures; however, while some neutral boron clusters do have quasi-planar or planar structures, others have tube or cage-like structures. The structures of boron clusters change after atom doping. In order to facilitate understanding via visualization, the low-lying isomers of PB_n_^0/−^ (n = 3–17), along with their corresponding relative energy values, are displayed in Figures S1–S30, and the lowest energy structures of PB_n_^0/−^ (n = 3–17) are depicted in Figure 1 and Figure 2. Clear structural diagrams show that the lowest-lying energy structures of PB_n_^−^ (n = 3–17) clusters have planar or quasi-planar structures, except for PB_17_^−^. The lowest energy structures of PB_n_ (n = 3–17) are planar or quasi-planar, with the exceptions of PB_7_, PB_9_, and PB_15_. As can be observed in Figure 1 and Figure 2, after the addition of the P atom, most boron clusters remain virtually unchanged from their corresponding bare boron cluster forms, such as PB_n_ (n = 3–6, 8, 10, 12) and PB_n_^−^ (n = 3, 5–8, 10–16) [23,50,51]. In order to further discuss the structural changes of boron clusters, we also compared their structures after replacing a boron atom with a phosphorus atom, and without replacing the boron atom. Compared with the bare boron clusters B_n_^0/−^ (n = 4–18), the structures of most PB_n−1_^0/−^ (n = 4–18) clusters changed, except for those of PB_3_^−^, PB_3_, PB_4_, and PB_5_ (which remained almost similar to those of B_4_^−^, B_4_, B_5_, and B_6_, respectively); that is, the structures of the bare boron clusters changed due to the replacement of one atom with a P atom. The lowest energy configurations of PB_n_^0/−^ (n = 3, 5, 6, 8) are planar structures, with the P atom attached to the boron atoms of the bare boron clusters B_n_^0/−^ (n = 3, 5, 6, 8) [50]. The lowest-lying energy structures of PB_n_^0/−^ (n =10, 12) are quasi-planar, similar to the ground-state structures of their corresponding bare boron clusters, B_10_^0/−^ and B_12_^0/−^, with the addition of a phosphorus atom bonded to the boron atoms. PB_10_^−^ has a chiral symmetrical structure. Likewise, the planar clusters PB_4_, PB_7_^−^, PB_11_^−^, PB_13_^−^, and PB_14_^−^, as well as the quasi-planar cluster PB_16_^−^, have similar structures to those of their corresponding bare boron clusters [23,50]. However, the ground-state structures of the planar PB_4_^−^ and quasi-planar PB_11_, PB_13_, and PB_16_ clusters are different from their corresponding bare boron clusters due to the action of the P atom.

The planar wheel-shaped structure of B_9_ becomes the three-dimensional structure of PB_9_ with the doping of a P atom; the anion cluster B_9_^−^, with a similar structure, changes from the planar wheel to the double-chain planar structure of PB_9_^−^. The lowest energy configurations of PB_14_ and PB_17_ have quasi-two-dimensional structures, and PB_17_^−^ has a three-dimensional cage-shaped structure. It is important to note the specific behaviors of PB_7_, PB_9_^−^, and PB_13_. For the PB_9_^−^ cluster, at the PBE0/6-311+G(d) level, the double-chain planar structure (Figure S14a) is more stable than the planar structure (Figure S14b), with an energy gap of 0.17 eV. At the higher level, CCSD(T)/6-311+G(d)//PBE0/6-311+G(d)+ZPE (zero-point energy corrections), both structures are almost degenerate in regards to energy, each with a tiny energy gap (that is, the planar structure (Figure S14b) is more stable than the double-chain planar structure (Figure S14a), with an energy gap of 0.01 eV). We consider the double-chain structure as the lowest energy structure of PB_9_^−^; the same is true for the PB_7_ and PB_13_ clusters. It is worth mentioning that the umbrella-like structure found in PB_7_ is also the lowest energy structure in many metal-doped boron clusters, such as LiB_7_, BeB_7_^0/−^, BeB_8_^0/−^, and MgB_8_ [52,53,54]. Research shows that the low-valent actinide(III) boron clusters AnB_7_ (An = Pa, U, Np, and Pu), with umbrella-shaped structures, exhibit high electronic stability and can be obtained in the gas phase at room temperature [55]. Similarly, the neutral cluster PB_7_ also has an umbrella-shaped structure, with C_6v_ symmetry. Therefore, in this study, we will highlight the umbrella-like structure of PB_7_, which can provide a theoretical basis for the design of highly stable boron-based nanomaterials. In addition, the quasi-planar structure of B_15_ becomes the three-dimensional half-sandwich-shaped structure of PB_15_ after P atom doping; thus, studying the structures and characteristics of PB_15_ is crucial to understanding the structural laws of doped nonmetallic boron clusters. In summary, the study of PB_7_ and PB_15_ is of great significance for the discovery of new stable boron-based nanomaterials. 

### 2.2. Relative Stabilities

**Figure 3:**
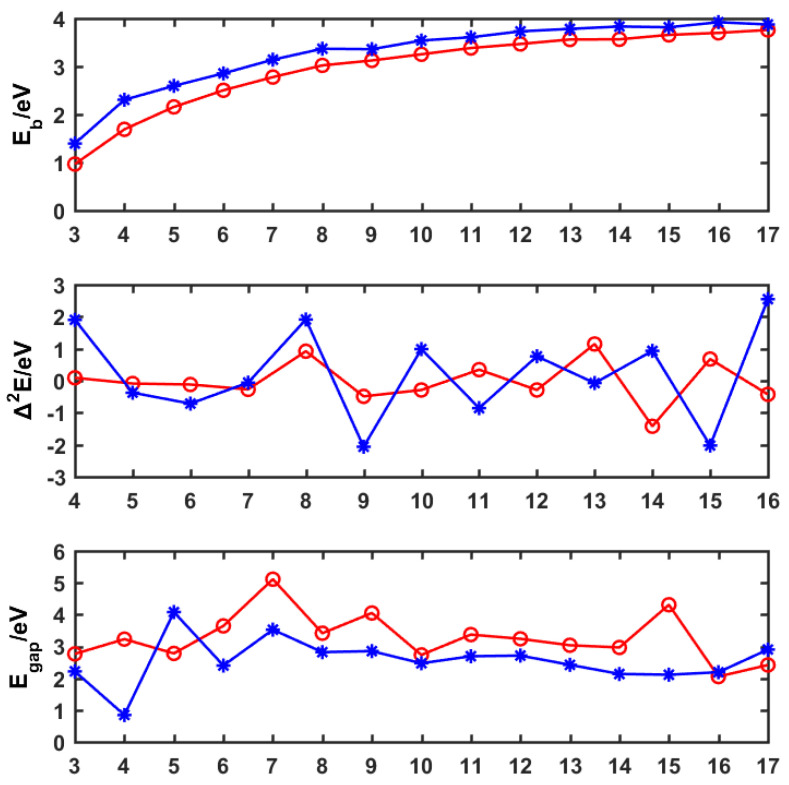
Average binding energy (*E*_b_), second-order energy differences (Δ^2^*E*), and HOMO–LUMO gaps (*E*_gap_) of doped boron clusters PB_n_^0/−^ (n = 3−17). Red represents the neutral cluster, blue represents the anion cluster, and n represents the number of boron atoms in the cluster.

To analyze the relative stabilities of the lowest energy level of PB_n_^0/−^ (n = 3−17), we calculated the average binding energy (*E*_b_) and the second-order energy differences (∆^2^*E*) of the clusters using the following formulas, where n represents the number of boron atoms, and *E* is the total energy of the corresponding atom or cluster. The calculation results obtained using Equations (1)–(3) are plotted in Figure 3. The formulas are as follows:

*E*_b_(PB_n_) = [n*E*(B) + *E*(P) − *E*(PB_n_)]/(n + 1) (1)

*E*_b_(PB_n_^−^) = [(n − 1)*E*(B) + *E*(P) + *E*(B^−^) − *E*(PB_n_^−^)]/(n + 1) (2)

Δ^2^*E*(PB_n_^0/−^) = *E*(PB_n−1_^0/−^) + *E*(PB_n+1_^0/−^) − 2*E*(PB_n_^0/−^) (3)

*E*_b_ represents the inherent stability of the cluster, in that a larger value of *E*_b_ denotes higher relative stability. As evidenced in Figure 3, the *E*_b_ values of PB_n_^0/−^ (n = 3−17) are gradually increasing with the increase in the boron atom number n, indicating that the cluster becomes more and more stable. Furthermore, the *E*_b_ values of the anions are overall more sizable than those of the corresponding neutral ions; thus, we can infer that the anion clusters of PB_n_^−^ (n = 3−17) are more stable than their corresponding neutral clusters, and that the excess electrons enhance the stability of the P-doped boron clusters. ∆^2^*E* is an important indicator of relative stability and can provide valuable insights into the stability of the clusters. In Figure 3, the second-order difference of the peaks found in n = 4, 8, 10, 12, 13, 14, 15, and 16 indicate that PB_4_^−^, PB_8_^−^, PB_8_, PB_10_^−^, PB_12_^−^, PB_13_, PB_14_^−^, PB_15_, and PB_16_^−^ are relatively more stable than their adjacent clusters.

The energy difference *E*_gap_ between HOMO and LUMO is an indicator of chemical stability. A larger *E*_gap_ value for a cluster implies that the electrons are more difficult to excite from HOMO to LUMO, which indicates that the corresponding structures are chemically inert. As can be seen in Figure 3, two obvious *E*_gap_ peaks are found in PB_7_ (about 5.11 eV) and PB_15_ (about 4.31 eV), implying that the neutral clusters PB_7_ and PB_15_ exhibit better chemical stability than that of the other clusters. Therefore, combining ∆^2^*E* and *E*_gap_ analyses proved that PB_15_ has a high relative and chemical stability. Additionally, due to the PB_7_ cluster having the largest values of *E*_gap_, as well as a special umbrella structure, we chose these clusters as examples through which to analyze chemical bonding in P-doped boron clusters.

### 2.3. Chemical Bonding Analysis

**Figure 4:**
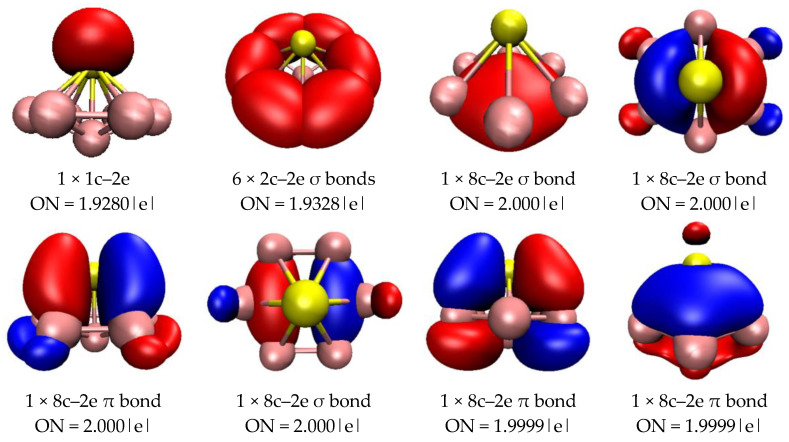
AdNDP analysis of PB_7_. ON is the occupation number, and the yellow ball is the P atom.

**Figure 5:**
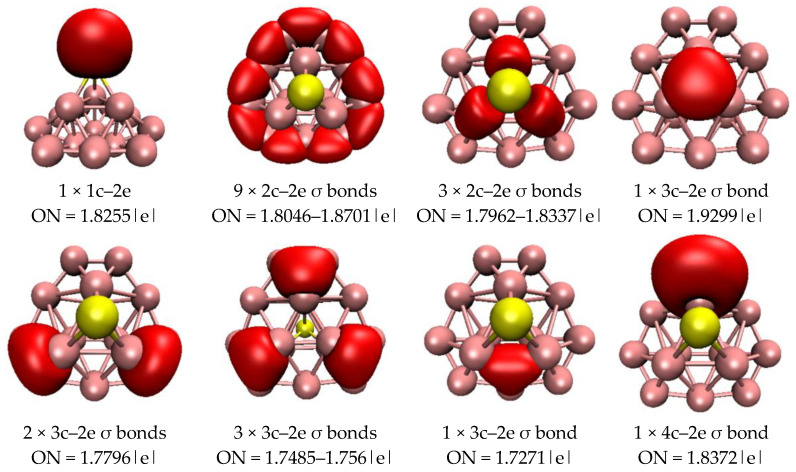

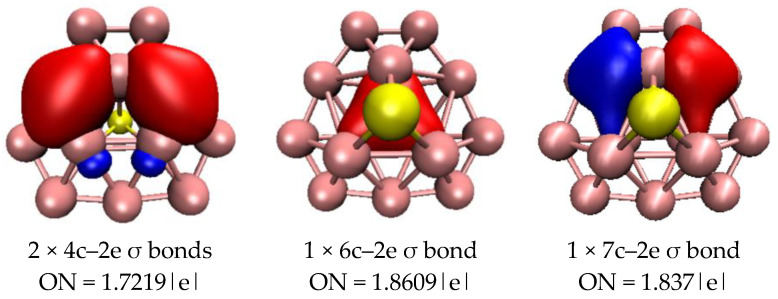
AdNDP analysis of PB_15_. ON is the occupation number, and the yellow ball is the P atom.

Through a comprehensive stability analysis of all of the clusters detailed in the previous section, we inferred that PB_7_ and PB_15_ show chemical stability, due to their high E_gap_ values. We analyzed the charge populations of PB_7_ and PB_15_ (see Figure S33). Different from the metal elements in metal-doped boron clusters, which can undergo one or more electron transfers, the phosphorus atom in PB_7_ or PB_15_ transfers less than one electron. To further understand the electronic properties and stability of the closed-shell clusters PB_7_ and PB_15_, the AdNDP method was used to analyze their chemical bonding characteristics. The AdNDP method is an extension of the popular natural bond orbital (NBO) analysis, which can be used to analyze localized and delocalized multicenter bonds. We quantitatively analyzed the bonding properties of PB_7_ and PB_15_ using the AdNDP method, and the results are depicted in Figure 4 and Figure 5. In Figure 4, we clearly observe six two-center two-electron (2c–2e) σ bonds around the peripheral B atoms of the PB_7_ cluster. In addition, there are three 8c–2e σ bonds and three 8c–2e π bonds in PB_7_, which together support the plane of the cluster and enhance the stability of PB_7_. As can be seen in the Figure 4, the contribution of the P atom to the 8c–2e π bonds is greater than its contribution to the 8c–2e σ bonds. The delocalized σ and π bonds give rise to double aromaticity and fulfill the Hückel 4N + 2 rule with N = 1.

For PB_15_ (Figure 5), there is one lone pair on the P atom, and three 2c–2e σ bonds cover the B–P bond attached to the P atom. These three 2c–2e σ bonds are formed by three electrons on the P atom combining with an electron of each B atom on the B_3_ ring. In addition, nine 2c–2e σ bonds are distributed over the peripheral B–B bond. The seven 3c–2e σ bonds are divided into the four following groups: one 3c–2e σ bond is distributed on the inner ring triangle at the back side of the P atom, two 3c–2e σ bonds are distributed on two peripheral B_3_ rings, three 3c–2e σ bonds cover the three peripheral B_3_ rings behind the P atom, and the last 3c–2e σ bond is distributed on a ternary boron ring. Moreover, there are three 4c–2e σ bonds over three B_4_ rings, one 6c–2e σ bond attached to an internal B_6_ ring, and one 7c–2e σ bond over a B_7_ ring. On the whole, there are strong interactions between B_15_ and the P atom through delocalized bonds that stabilize the PB_15_ cluster. According to AdNDP investigations, the PB_15_ cluster possesses 12 delocalized σ bonds that do not satisfy spherical aromaticity [2(n + 1)^2^ rule].

**Figure 6:**
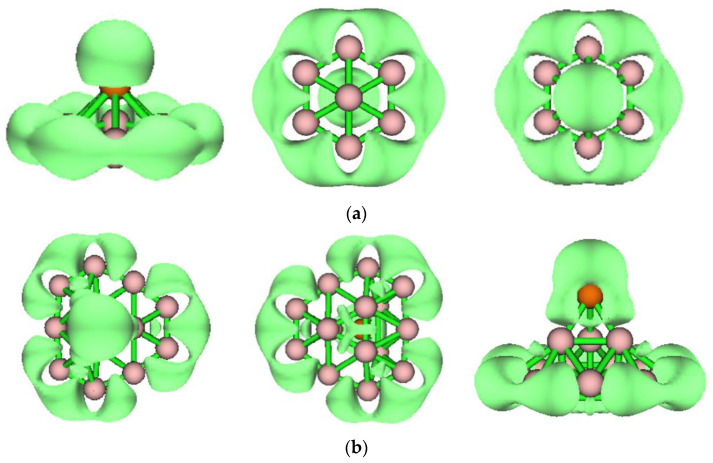
ELF at the PBE0 level. (**a**) C_6v_ PB_7_; the isovalue is 0.8. (**b**) C_S_ PB_15_; the isovalue is 0.8.

To further confirm the above AdNDP analysis results, we analyzed PB_7_ and PB_15_ using the ELF method, a function that can be used to describe the localization and delocalization of different molecular regions. The ELF results for PB_7_ and PB_15_ are shown in Figure 6, as well as in Figures S31 and S32, respectively. As can be observed from Figure 6 and Figure S31, the isosurface map of PB_7_ covers six peripheral B–B bonds that correspond to six peripheral 2c–2e σ bonds, as well as the entire region of B and P atoms corresponding to the 8c–2e bonds. Combining Figure S32 and Figure 6 shows that the isosurface map of PB_15_ covers nine peripheral B–B bonds and three B–P bonds, which correspond to twelve 2c–2e σ bonds, as well as seven B_3_ triangles that correspond to seven 3c–2e σ bonds. Additionally, the three regions around PB_15_ are fatter, indicating the presence of 3c–2e and 4c–2e bonds.

### 2.4. Photoelectron Spectra

**Figure 7:**
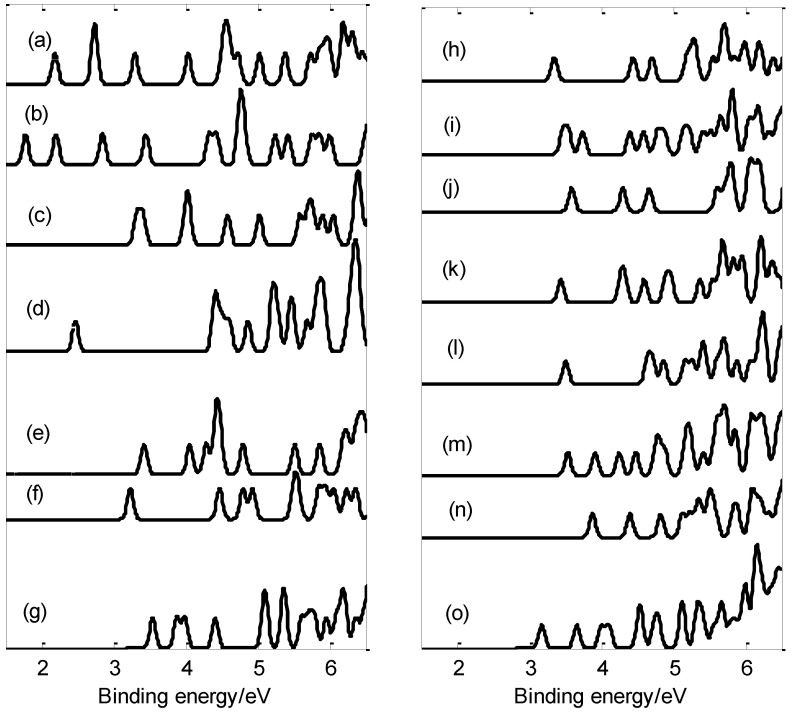
Photoelectron spectra at the PBE0/6-311+G * level: (**a**) PB_3_^−^; (**b**) PB_4_^−^; (**c**) PB_5_^−^; (**d**) PB_6_^−^; (**e**) PB_7_^−^; (**f**) PB_8_^−^; (**g**) PB_9_^−^; (**h**) PB_10_^−^; (**i**) PB_11_^−^; (**j**) PB_12_^−^; (**k**) PB_13_^−^; (**l**) PB_14_^−^; (**m**) PB_15_^−^; (**n**) PB_16_^−^; (**o**) PB_17_^−^.

Photoelectron spectra (PES) is an effective approach for exploring the energy levels of valence electrons in nanoclusters. In photoelectron spectra, the positions of the peaks represent the energy differences between the initial and final electronic state after photons absorption. In order to identify the structures of the PB_n_^−^ (n = 3−17) clusters, we calculated the vertical detachment energies (VDEs) of the anionic clusters and simulated the photoelectron spectra of the PB_n_^−^ (n = 3−17) clusters using the time-dependent density functional theory (TD-DFT). The first few peaks of the photoelectron spectrum are commonly used to identify boron clusters [10,15]; thus, studying the peaks on the low-binding-energy side is of significant importance. Figure 7 shows the photoelectron spectra of the PB_n_^−^ (n = 3–17) clusters. According to the photoelectron spectra, the anion PB_16_^−^ exhibits the largest first VDE value (3.88 eV, list in Table S1), while PB_4_^−^ shows the lowest first VDE (1.78 eV). In addition, the energy gap (about 1.95 eV) between the first and second PB_6_^−^ energy bands is the largest.

The first peaks of the photoelectron spectrum (except for those of PB_4_^−^, PB_5_^−^ and PB_11_^−^) are derived from the calculated ground-state VDEs of PB_3_^−^, PB_6_^−^, PB_7_^−^, PB_8_^−^, PB_9_^−^, PB_10_^−^, PB_12_^−^, PB_13_^−^, PB_14_^−^, PB_15_^−^, PB_16_^−^ and PB_17_^−^ at 2.19, 2.47, 3.43, 3.24, 3.55, 3.35, 3.59, 3.44, 3.51, 3.53, 3.88, and 3.17 eV, respectively. The first peak of PB_4_^−^ comes from the second VDE (2.2 eV). For open-shell PB_5_^−^ and PB_11_^−^, their first peaks come from the first and second VDEs (3.33 and 3.41 eV for PB_5_^−^, 3.47 and 3.54 eV for PB_11_^−^). Furthermore, the second peaks of PB_7_^−^, PB_8_^−^, PB_9_^−^, PB_10_^−^, PB_12_^−^, PB_14_^−^, PB_15_^−^, PB_16_^−^, and PB_17_^−^ come from the second VDEs at 3.72, 4.06, 4.47, 3.88, 4.44, 4.3, 4.64, 3.9, 4.4, and 3.66 eV, respectively. The second peak of the photoelectron spectrum of PB_4_^−^ comes from the ground-state VDE at 1.78 eV. However, the second peaks of PB_3_^−^, PB_6_^−^, and PB_13_^−^ are derived from the second and third VDEs (2.736 and 2.744 eV for PB_3_^−^; 4.4 and 4.43 eV for PB_6_^−^; 4.27 and 4.33 eV for PB_13_^−^). The second peak of PB_5_^−^ comes from the third VDE (4.01 eV) and the fourth VDE (4.05 eV). For PB_11_^−^, the second peak comes from the third VDE (3.74 eV). Furthermore, peaks with higher binding energies are derived from the separation of electrons from lower molecular orbitals.

Partially anionic boron clusters doped with P atoms have similar structures to those of the corresponding bare boron clusters; however, a comparison of their photoelectron spectra revealed that the addition of a phosphorus atom results in large changes in the photoelectron spectra of most clusters (except for PB_11_^−^) [23,50]. For instance, the doped P atom causes the first peaks of PB_3_^−^, PB_6_^−^, and PB_13_^−^ to move 0.63, 0.54, and 0.34 eV, respectively, towards the low-binding-energy side. At the same time, the first peaks of PB_5_^−^, PB_7_^−^, PB_8_^−^, PB_10_^−^, PB_12_^−^, PB_14_^−^, PB_15_^−^, and PB_16_^−^ moved 0.93, 0.58, 0.22, 0.29, 1.33, 0.41, 0.1, and 0.49 eV, respectively, to the high-binding-energy side. As can be seen from Figure 7, all photoelectron spectra are different, which indicates that the addition of P atoms not only alters the geometric structure of the clusters, but also leads to changes in their electronic structures. In summary, it is of great significance to study the peaks at the low-binding-energy side, as these simulated spectra may be used as fingerprints with which to identify PB_n_^−^ (n = 3–17) structures in the future.

## 3. Computation Details

In this paper, the geometrical structure searches for the neutral and anionic clusters of PB_n_^0/−^ (n = 3–17) were performed using CALYPSO, an efficient and reliable method for searching for geometrical cluster configurations which has been successfully applied to the study of both boron clusters and doped boron clusters [25,27,33,36,40,54,56,57,58,59]. CALYPSO 5.0 software generates 70% of the structure in each generation, and the remaining 30% is formed randomly. When the size of a boron atom is in the range of n = 3–10, nearly 100–1200 isomers are initially obtained for each cluster. The number of isomers increases with the number of boron atoms; when n = 11–14, there are approximately 2000 isomers for each cluster, and when n = 15–17, the number of isomers increases to 2500.

After the initial structural search, the lower energy structures of PB_n_^0/−^ (n = 3–17) were optimized at the PBE0/6-311+G(d) level, which is a reliable level for analyzing boron clusters, since the theoretical simulation values (photoelectron spectra) are consistent with the experimental values [15,25,38,59,60,61]. In order to obtain more accurate relative energy values, we performed CCSD(T) [62] calculations [CCSD(T)/6-311+G(d)//PBE0/6-311+G(d)]+ZPE(zero-point energy corrections) using the optimized PBE0 geometries for the collected isomers. The harmonic frequency and electronic structures were analyzed at the PBE0/6-311+G(d) level; therefore, the calculations below were obtained using the PBE0/6-311+G(d) and CCSD(T)/6-311+G(d)//PBE0/6-311+G(d)+ZPE methods, employing Gaussian16 software [63]. In addition, AdNDP and ELF are implemented in Multiwfn 3.7 software [64], and the AdNDP results were visualized using Visual Molecular Dynamics (VMD) 1.9.3 software [65].

## 4. Conclusions

In this work, the ground state structures of P-doped boron clusters PB_n_^0/−^ (n = 3–17) were identified using the CALYPSO method. In addition, the bonding properties of PB_7_ and PB_15_ were discussed, based on AdNDP and ELF analyses, and the photoelectron spectra of the anionic clusters were also calculated. The conclusions can be summarized as follows: (1) The lowest-lying energy structures of PB_n_^−^ (n = 3–17) clusters, except for PB_17_^−^, exhibit planar or quasi-planar structures. The lowest energy structures of PB_n_ (n = 3–17) are planar or quasi-planar, with the exceptions of PB_7_, PB_9_, and PB_15_. The lowest energy structure of PB_7_ has an umbrella-like structure, with high symmetry (C_6V_), and the ground-state configuration of PB_10_^−^ exhibits a chiral symmetric structure. (2) The lowest energy structures of the neutral ion PB_15_ have a half-sandwich structure, and they possess relatively high ∆^2^*E* and *E*_gap_ values, indicating that they possess superior relative and chemical stability. (3) AdNDP bonding analysis and ELF analysis further verified the stability and validity of the lowest energy structures of PB_7_ and PB_15_. (4) The PB_n_^−^ anionic clusters (n = 3–17) exhibit different photoelectron spectra on their low-binding-energy sides, which can provide a theoretical basis for the identification of doped boron clusters.

## Data Availability

The data presented in this study are available in the article and the Supplementary Materials.

## Supplementary Materials

The following supporting information can be downloaded at: https://www.mdpi.com/article/10.3390/molecules29143384/s1, Figures S1–S30: Low-lying isomers of doped boron clusters PB_n_^0/−^ (n = 3–17); Figure S31: Electron localization function (ELF) of PB_7_ with the isovalue set to 0.59; Figure S32: Electron localization function (ELF) of PB_15_ with the isovalue set to 0.7; Figure S33: Charge population of PB_7_ and PB_15_; Table S1: The first VDE values of bare B clusters and P-doped boron clusters.
