# Supplementary material for: Structural Evolution of Small-Sized Phosphorus-Doped Boron Clusters: A Half-Sandwich-Structured PB15 Cluster
Source: Molecules. 2024 Jul 18;29(14):3384. doi: 10.3390/molecules29143384 (PMC11280394; doi:10.3390/molecules29143384)
Supplement: Supplementary file 1 [file molecules-29-03384-s001.zip › molecules-3058691-supplementary.pdf]

Structural Evolution of Small-Sized Phosphorus-Doped Boron Clusters: A Half-Sandwich-Structured PB_{15} Cluster

Danyu Wang, Yueju Yang, Shixiong Li *, and Deliang Chen

School of Physics and Electronic Science, Guizhou Education University, Guiyang 550018, China.

*Correspondence: E-mail:lishixiong@gznc.edu.cn

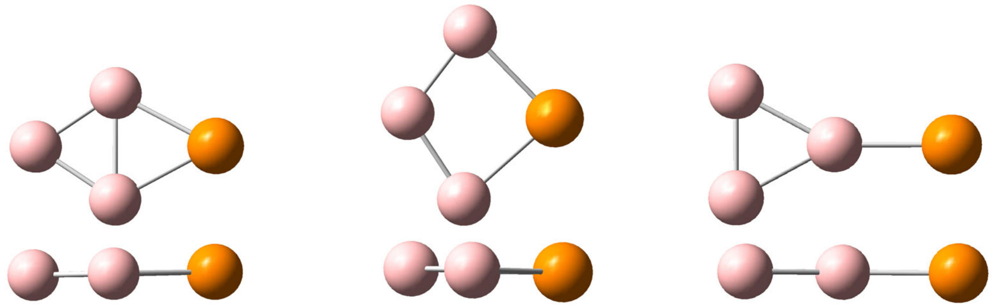

(a) [0] (0) (b) [0.61] (0.26) (c) [2.45] (2.01)

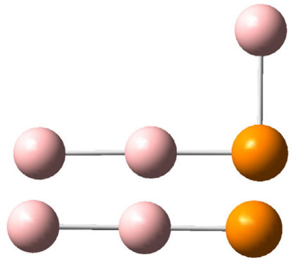

(d) [3.53] (2.87)

Figure S1. Low-lying isomers of doped boron clusters PB_3. Values in square brackets are the relative energies (eV) of the four low-energy isomers for PB_3 at the PBE0/6-311+G(d) level. Values in parentheses are the relative energies (eV) of the four low-energy isomers for PB_3 at the CCSD(T)/6-311+G(d)//PBE0/6-311+G(d) level. The upper row is top view and the bottom row is side view.

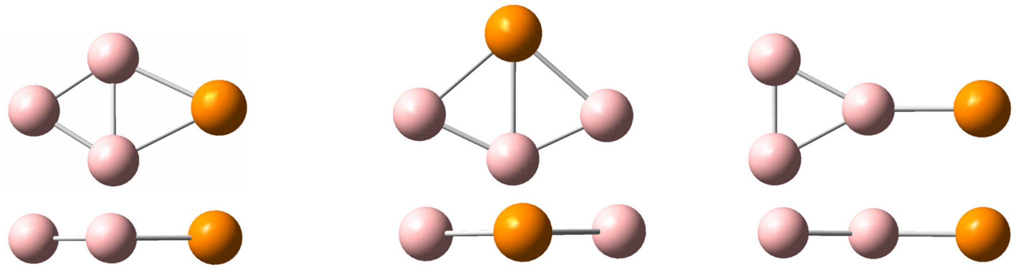

(a) [0] (0) (b) [0.58] (0.54) (c) [1.47] (1.58)

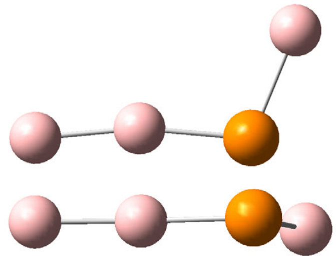

(d) [3.15]

Figure S2. Low-lying isomers of doped boron clusters PB_3^-. Values in square brackets are the relative energies (eV) of the four low-energy isomers for PB_3^- at the PBE0/6-311+G(d) level. Values in parentheses are the relative energies (eV) of the four low-energy isomers for PB_3^- at the CCSD(T)/6-311+G(d)//PBE0/6-311+G(d) level. The upper row is top view and the bottom row is side view.

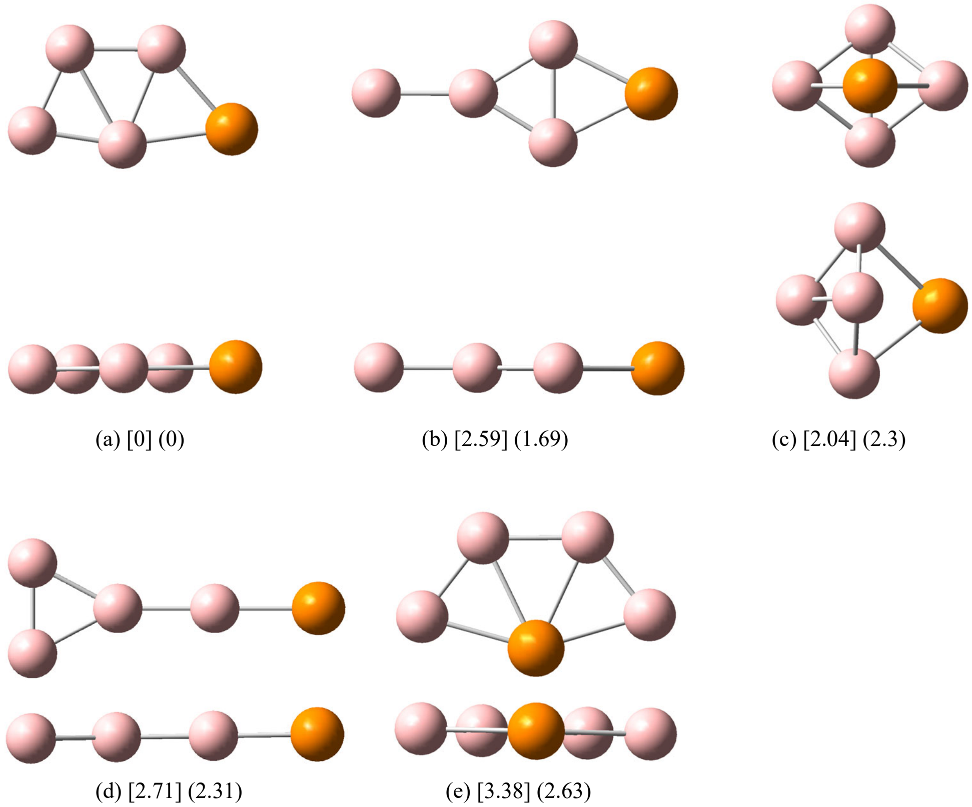

Figure S3. Low-lying isomers of doped boron clusters PB_4. Values in square brackets are the relative energies (eV) of the five low-energy isomers for PB_4 at the PBE0/6-311+G(d) level. Values in parentheses are the relative energies (eV) of the five low-energy isomers for PB_4 at the CCSD(T)/6-311+G(d)//PBE0/6-311+G(d) level. The upper row is top view and the bottom row is side view.

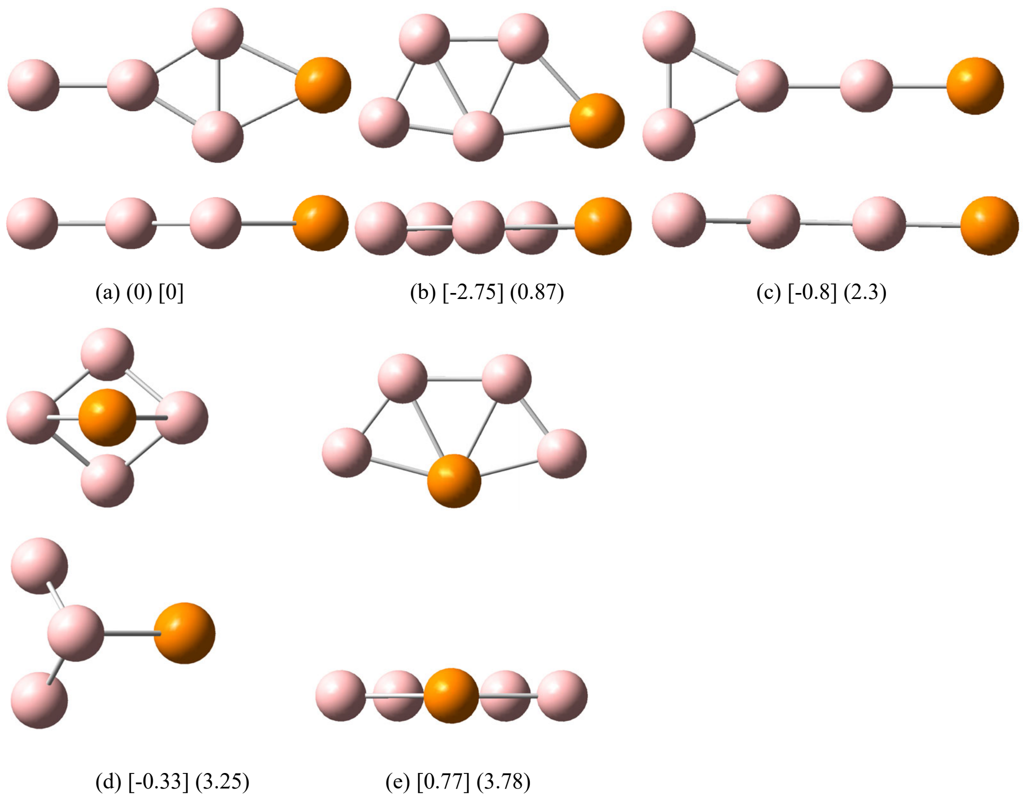

Figure S4. Low-lying isomers of doped boron clusters PB_4^-. Values in square brackets are the relative energies (eV) of the five low-energy isomers for PB_4^- at the PBE0/6-311G(d) level. Values in parentheses are the relative energies (eV) of the five low-energy isomers for PB_4^- at the CCSD(T)/6-311+G(d)//PBE0/6-311+G(d) level. The upper row is top view and the bottom row is side view.

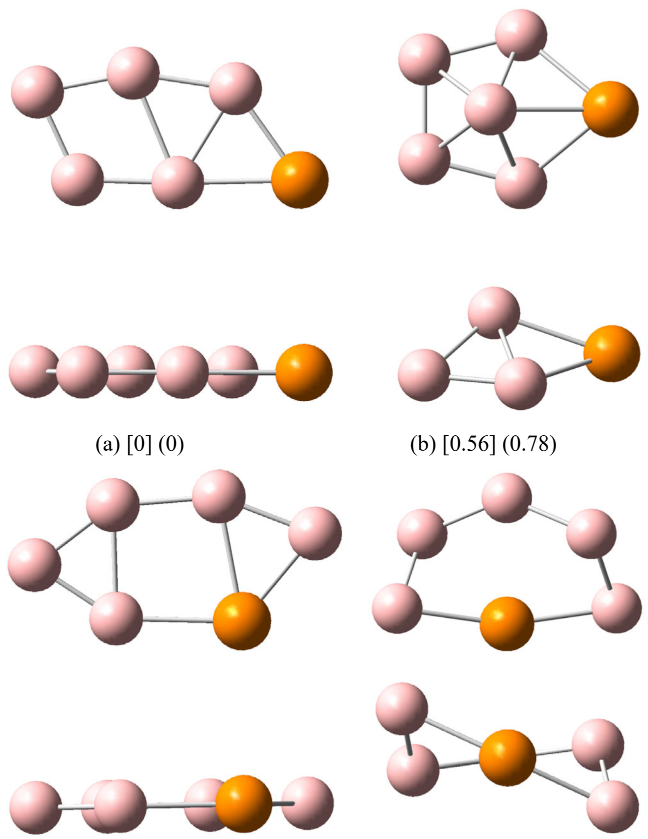

(a) [0] (0) (b) [0.56] (0.78) (c) [1.21] (1.2)

(d) [3.09] (2.58) (e) [3.58] (3.17)

Figure S5. Low-lying isomers of doped boron clusters PB_5. Values in square brackets are the relative energies (eV) of the five low-energy isomers for PB_5 at the PBE0/6-311+G(d) level. Values in parentheses are the relative energies (eV) of the five low-energy isomers for PB_5 at the CCSD(T)/6-311+G(d)//PBE0/6-311+G(d) level. The upper row is top view and the bottom row is side view.

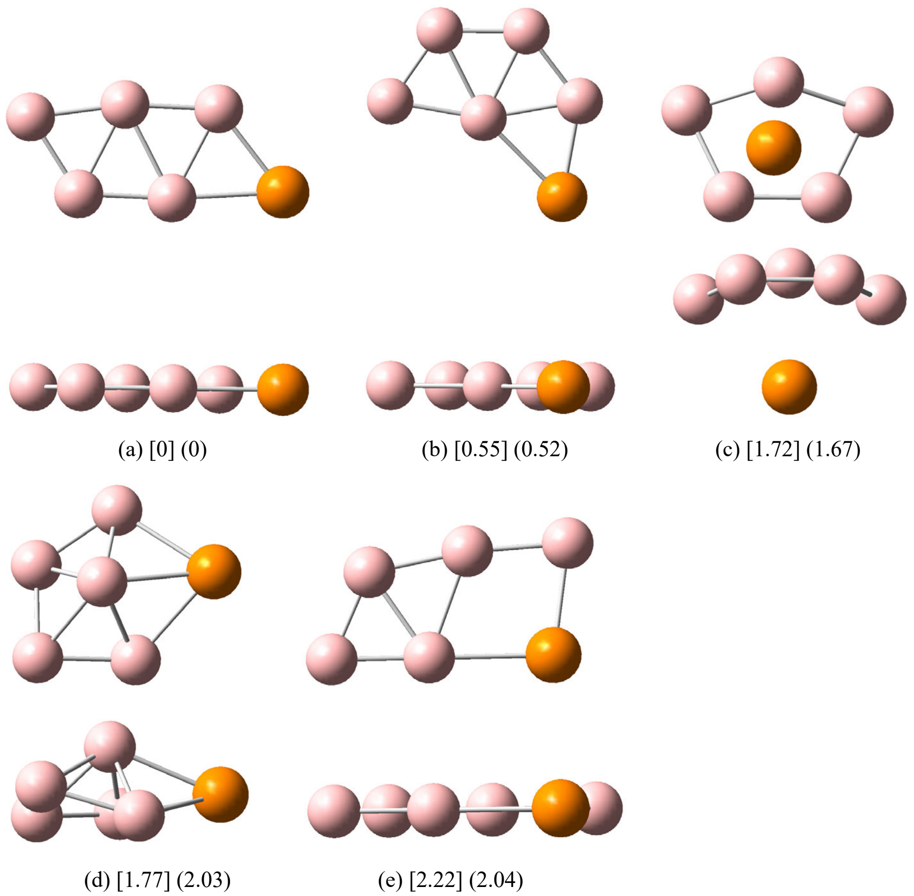

FigureS6. Low-lying isomers of doped boron clusters PB_5^-. Values in square brackets are the relative energies (eV) of the five low-energy isomers for PB_5^- at the PBE0/6-311+G(d) level. Values in parentheses are the relative energies (eV) of the five low-energy isomers for PB_5^- at the CCSD(T)/6-311+G(d)//PBE0/6-311+G(d) level. The upper row is top view and the bottom row is side view.

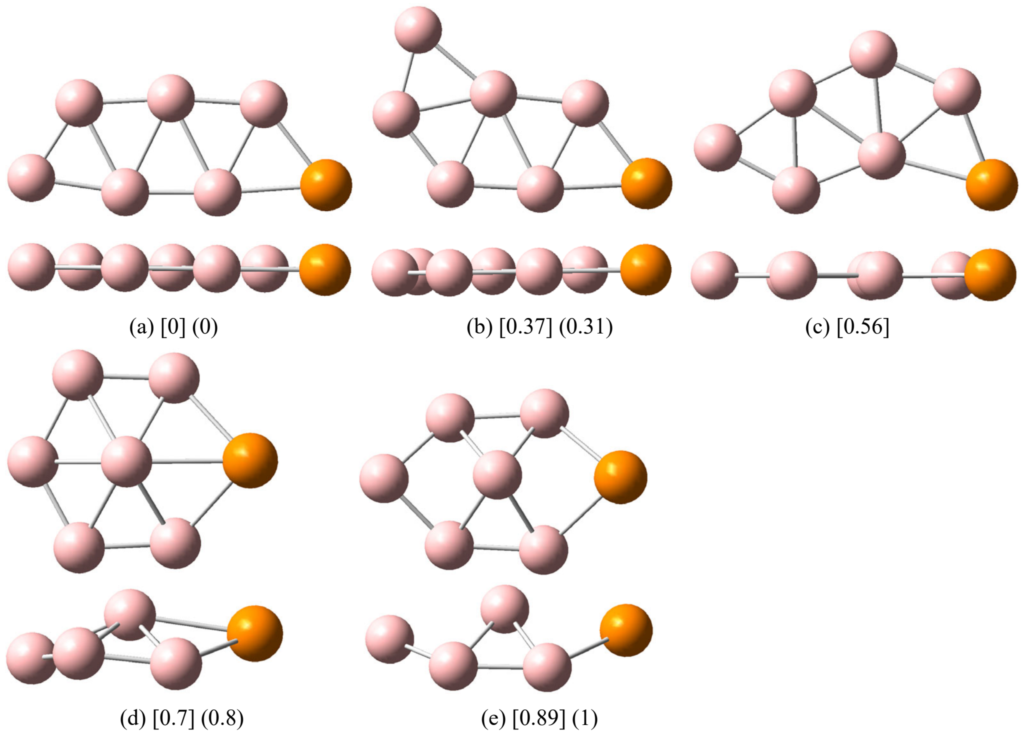

Figure S7. Low-lying isomers of doped boron clusters PB_6. Values in square brackets are the relative energies (eV) of the five low-energy isomers for PB_6 at the PBE0/6-311+G(d) level. Values in parentheses are the relative energies (eV) of the five low-energy isomers for PB_6 at the CCSD(T)/6-311+G(d)//PBE0/6-311+G(d) level. The upper row is top view and the bottom row is side view.

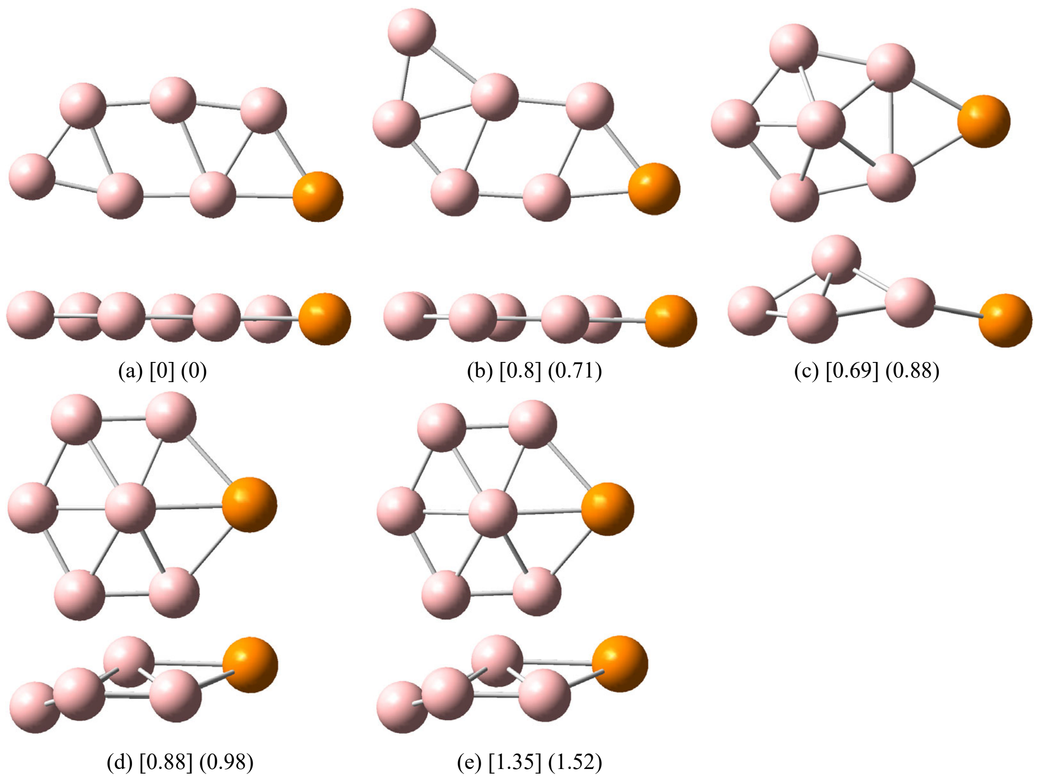

Figure S8. Low-lying isomers of doped boron clusters PB_6^-. Values in square brackets are the relative energies (eV) of the five low-energy isomers for PB_6^- at the PBE0/6-311+G(d) level. Values in parentheses are the relative energies (eV) of the five low-energy isomers for PB_6^- at the CCSD(T)/6-311+G(d)//PBE0/6-311+G(d) level. The upper row is top view and the bottom row is side view.

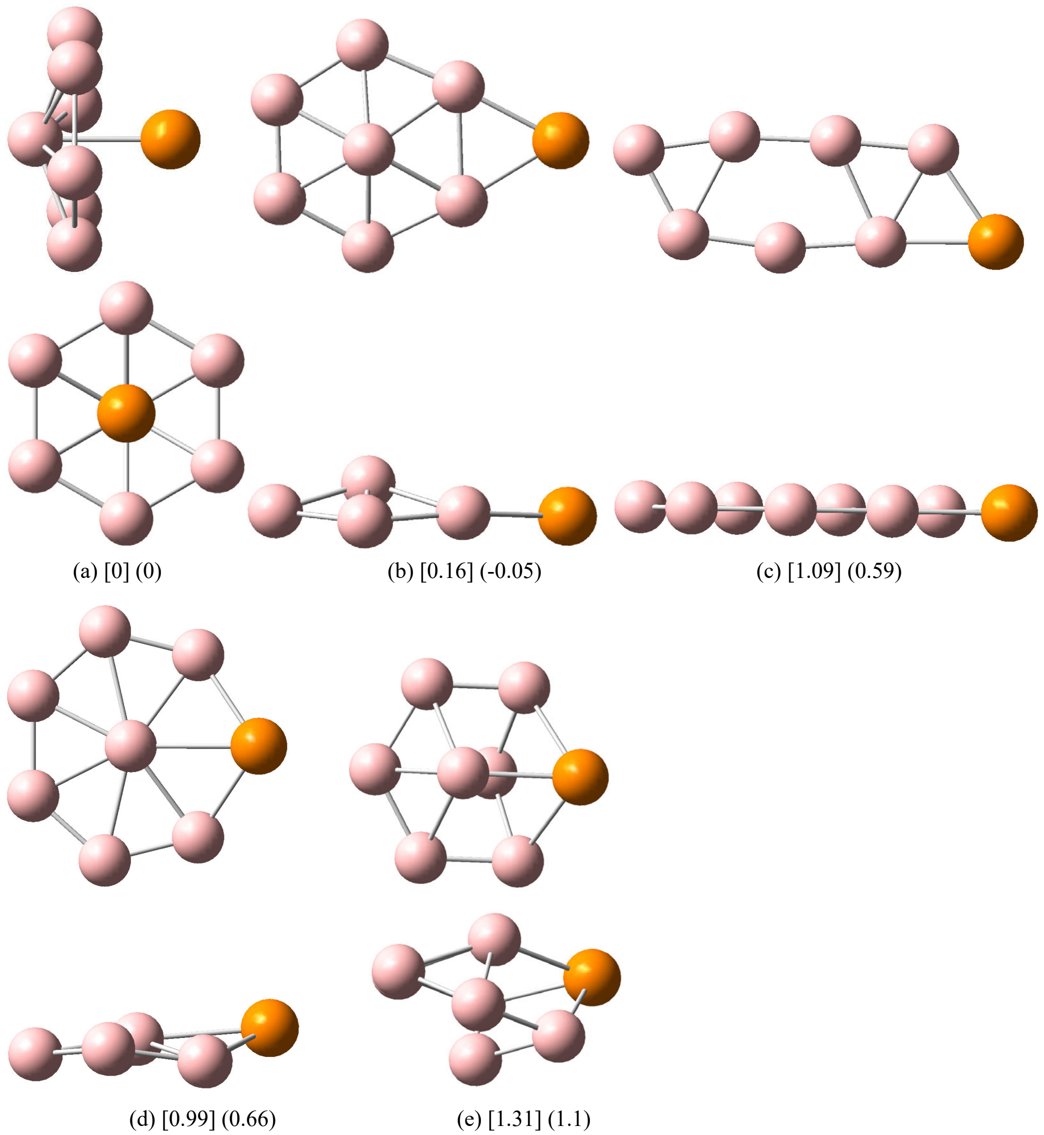

Figure S9. Low-lying isomers of doped boron clusters PB_7. Values in square brackets are the relative energies (eV) of the five low-energy isomers for PB_7 at the PBE0/6-311+G(d) level. Values in parentheses are the relative energies (eV) of the five low-energy isomers for PB_7 at the CCSD(T)/6-311+G(d)//PBE0/6-311+G(d) level. The upper row is top view and the bottom row is side view.

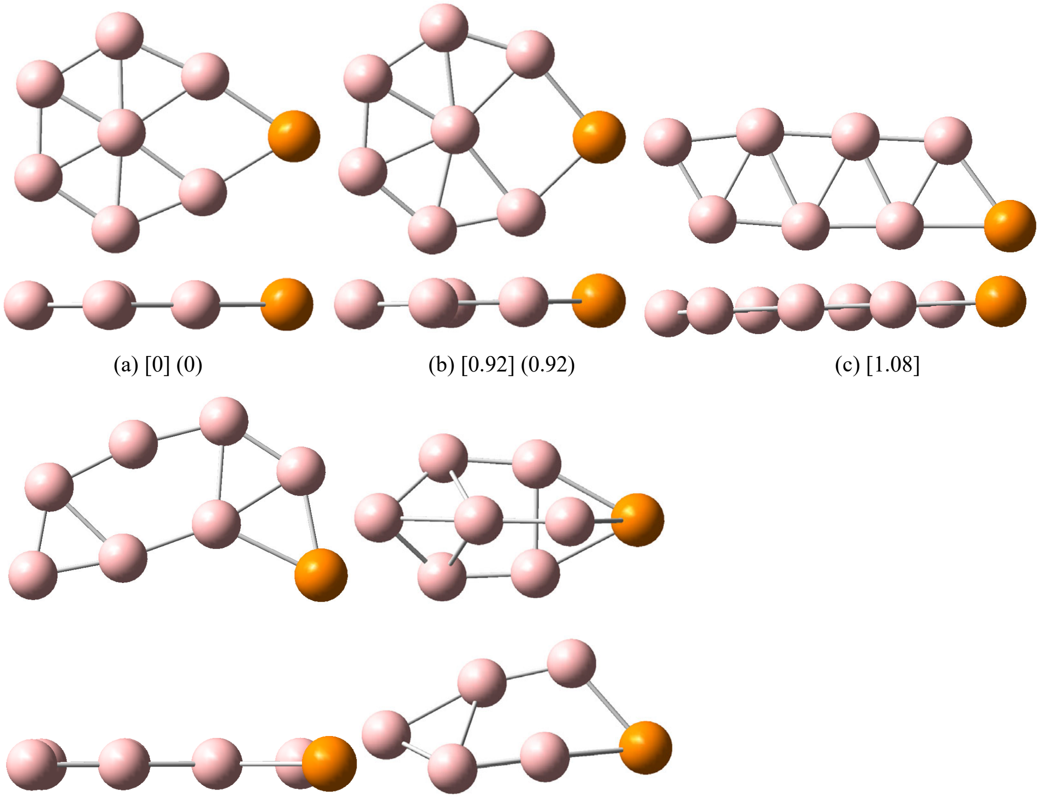

(a) [0] (0) (b) [0.92] (0.92) (c) [1.08]

(d) [1.45] (1.22) (e) [1.41] (1.45)

Figure S10. Low-lying isomers of doped boron clusters PB_7^-. Values in square brackets are the relative energies (eV) of the five low-energy isomers for PB_7^- at the PBE0/6-311+G(d) level. Values in parentheses are the relative energies (eV) of the five low-energy isomers for PB_7^- at the CCSD(T)/6-311+G(d)//PBE0/6-311+G(d) level. The upper row is top view and the bottom row is side view.

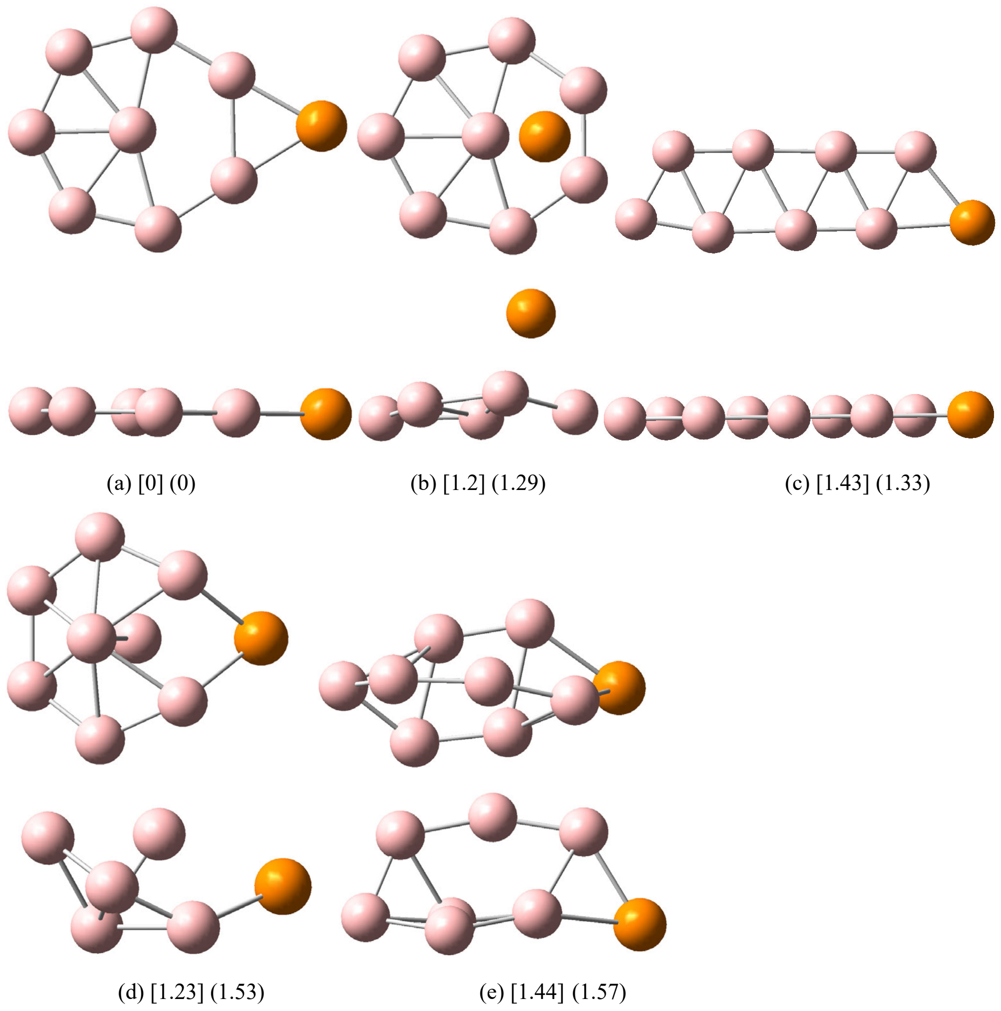

Figure S11. Low-lying isomers of doped boron clusters PB_8. Values in square brackets are the relative energies (eV) of the five low-energy isomers for PB_8 at the PBE0/6-311+G(d) level. Values in parentheses are the relative energies (eV) of the five low-energy isomers for PB_8 at the CCSD(T)/6-311+G(d)//PBE0/6-311+G(d) level. The upper row is top view and the bottom row is side view.

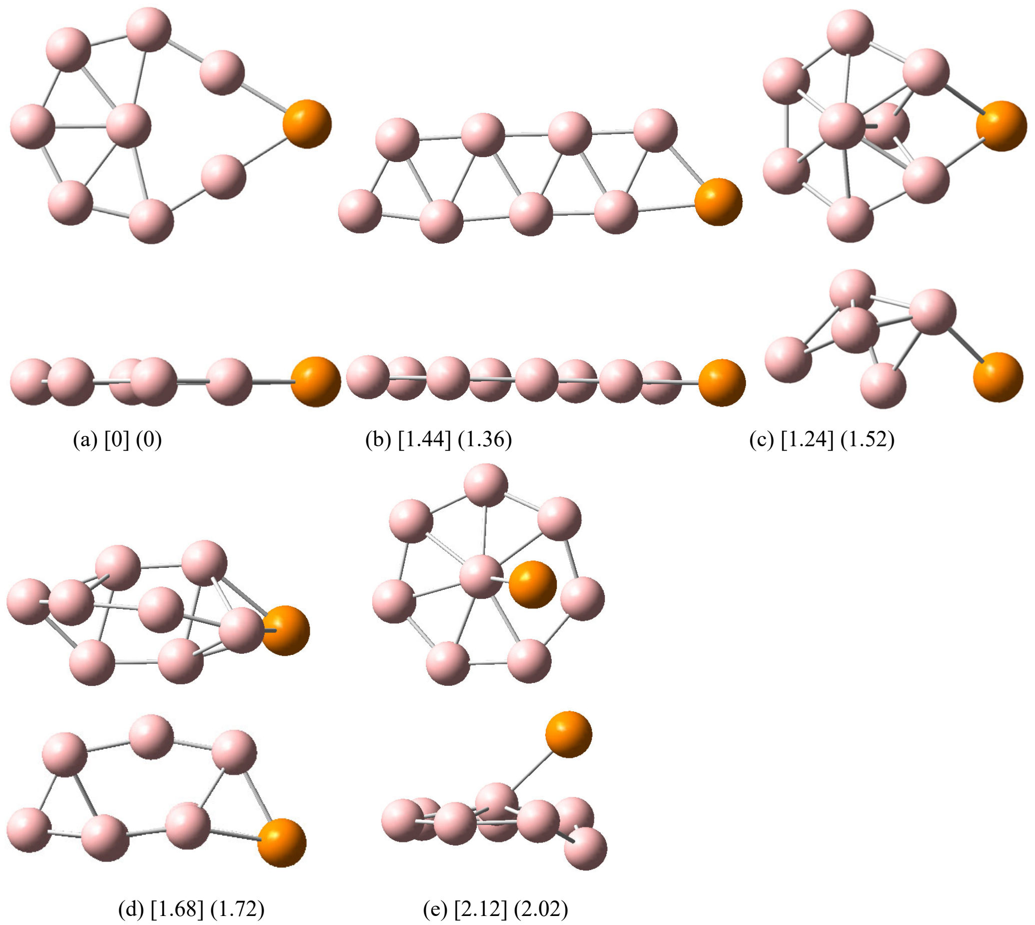

Figure S12. Low-lying isomers of doped boron clusters PB_8^-. Values in square brackets are the relative energies (eV) of the five low-energy isomers for PB_8^- at the PBE0/6-311+G(d) level. Values in parentheses are the relative energies (eV) of the five low-energy isomers for PB_8^- at the CCSD(T)/6-311+G(d)//PBE0/6-311+G(d) level. The upper row is top view and the bottom row is side view.

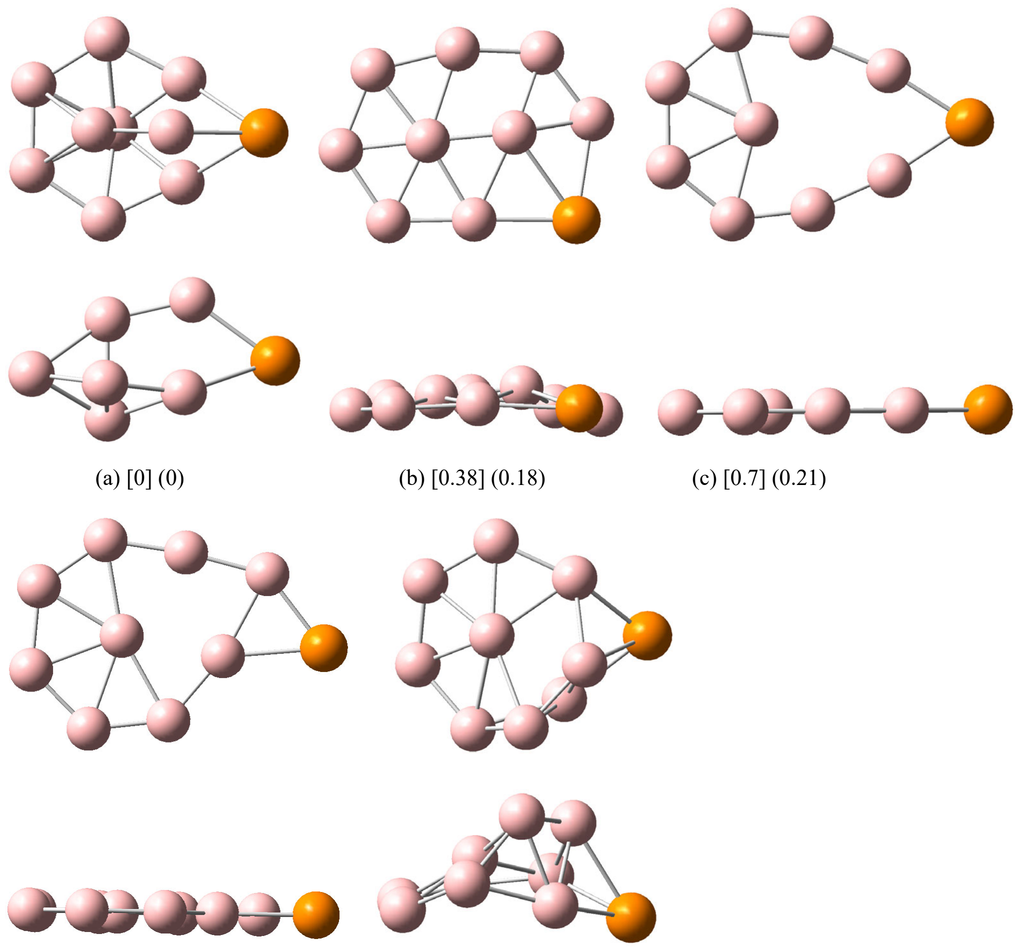

(a) [0] (0) (b) [0.38] (0.18) (c) [0.7] (0.21)

(d) [0.6] (0.22) (e) [0.71] (0.58)

Figure S13. Low-lying isomers of doped boron clusters PB_9. Values in square brackets are the relative energies (eV) of the five low-energy isomers for PB_9 at the PBE0/6-311+G(d) level. Values in parentheses are the relative energies (eV) of the five low-energy isomers for PB_9 at the CCSD(T)/6-311+G(d)//PBE0/6-311+G(d) level. The upper row is top view and the bottom row is side view.

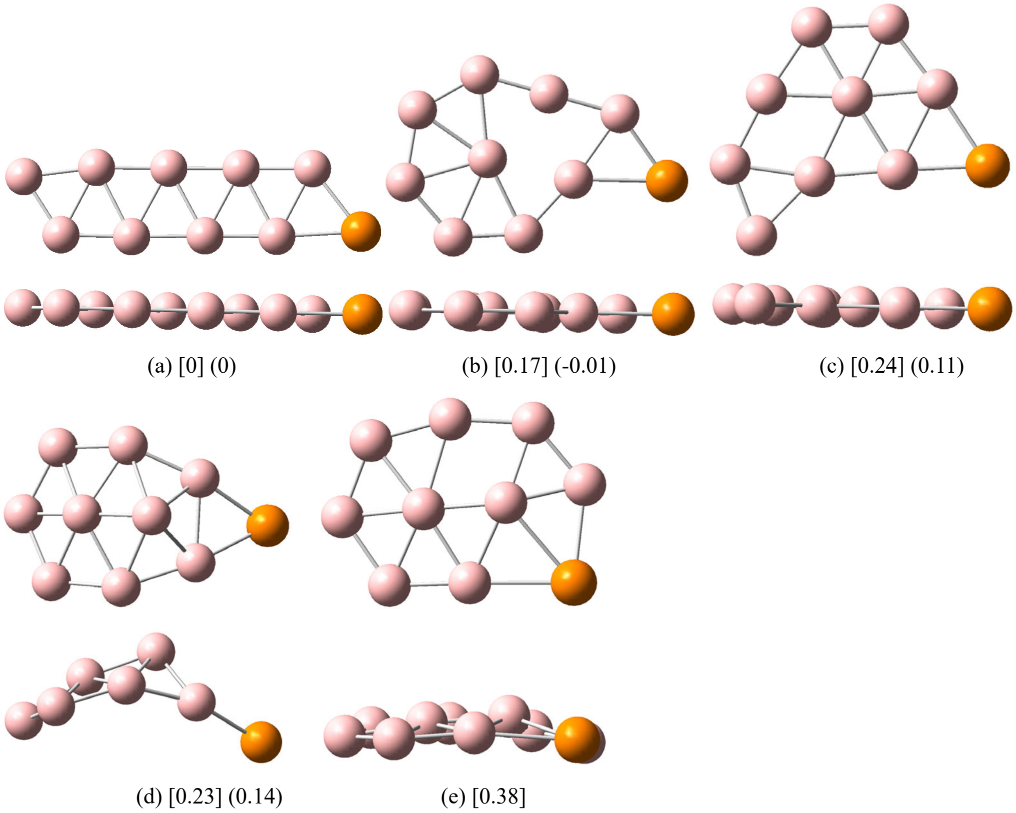

Figure S14. Low-lying isomers of doped boron clusters PB_9^-. Values in square brackets are the relative energies (eV) of the five low-energy isomers for PB_9^- at the PBE0/6-311+G(d) level. Values in parentheses are the relative energies (eV) of the five low-energy isomers for PB_9^- at the CCSD(T)/6-311+G(d)//PBE0/6-311+G(d) level. The upper row is top view and the bottom row is side view.

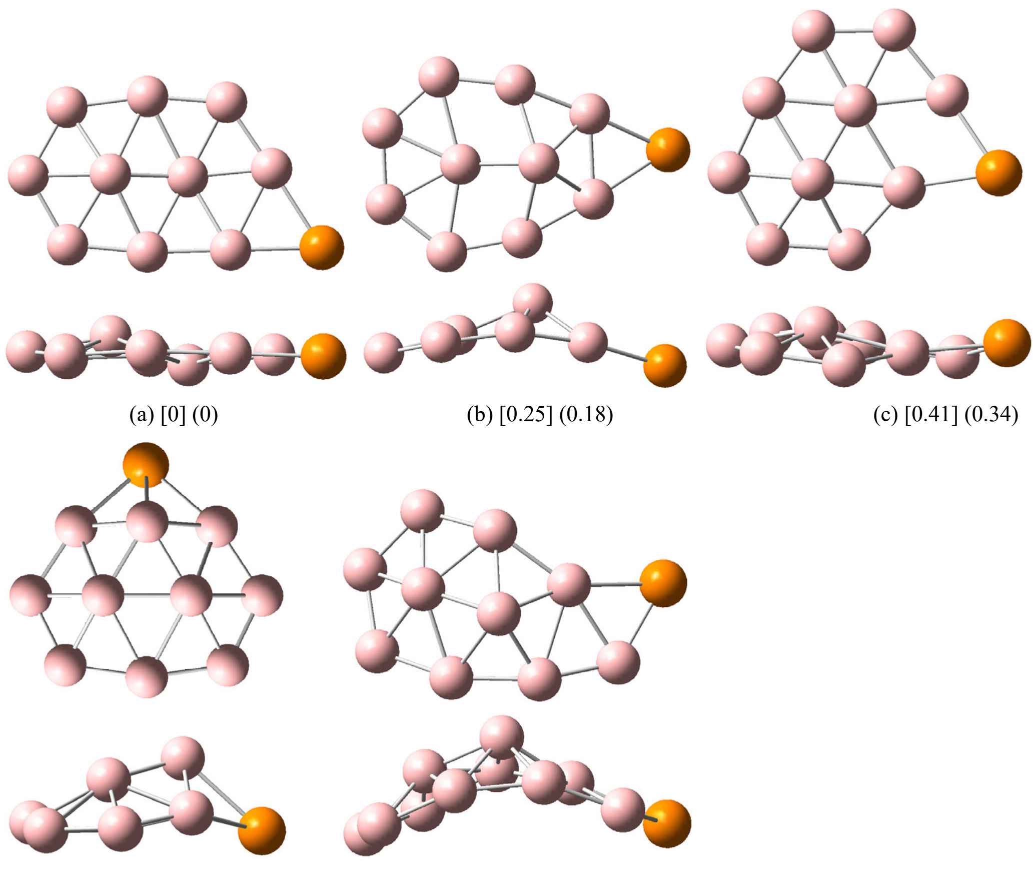

Figure S15. Low-lying isomers of doped boron clusters PB_{10}. Values in square brackets are the relative energies (eV) of the five low-energy isomers for PB_{10} at the PBE0/6-311+G(d) level. Values in parentheses are the relative energies (eV) of the five low-energy isomers for PB_{10} at the CCSD(T)/6-311+G(d)//PBE0/6-311+G(d) level. The upper row is top view and the bottom row is side view.

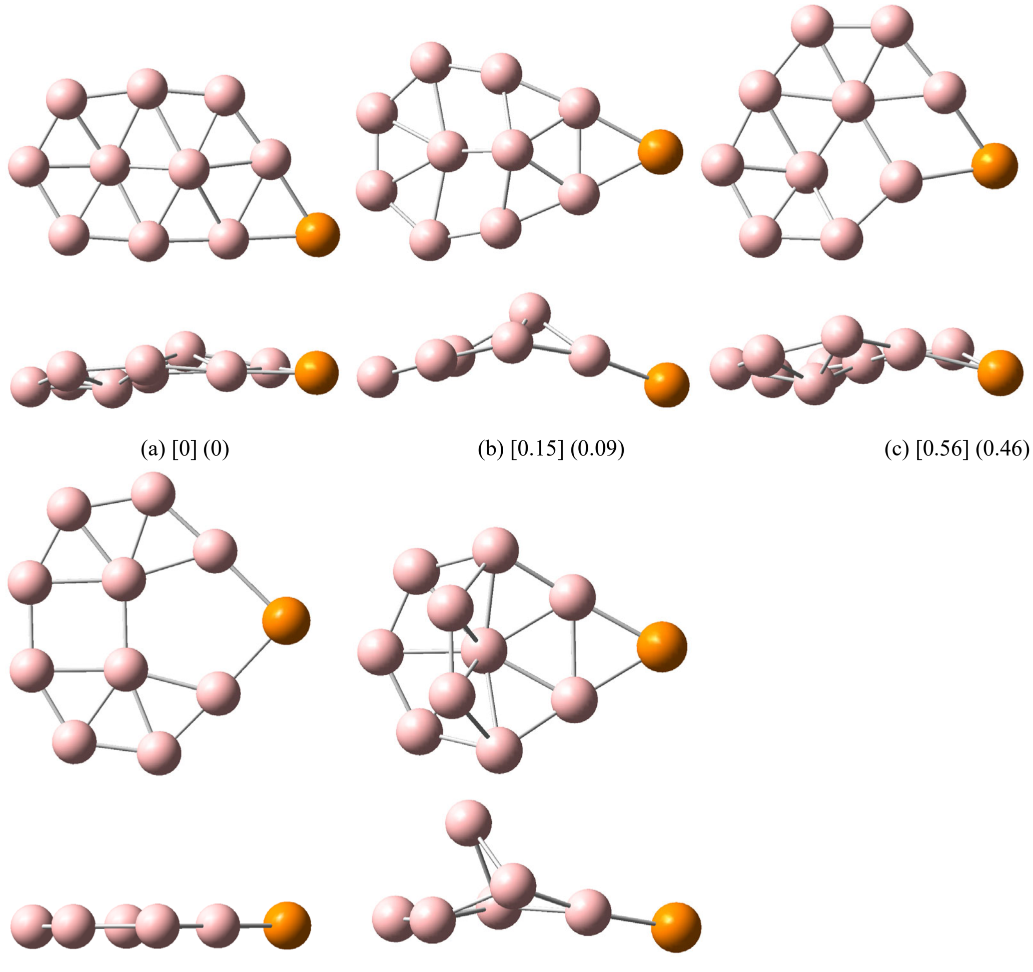

(a) [0] (0) (b) [0.15] (0.09) (c) [0.56] (0.46)

(d) [0.75] (0.48) (e) [0.82] (0.65)

Figure S16. Low-lying isomers of doped boron clusters PB_{10}^{-}. Values in square brackets are the relative energies (eV) of the five low-energy isomers for PB_{10}^{-} at the PBE0/6-311+G(d) level. Values in parentheses are the relative energies (eV) of the five low-energy isomers for PB_{10}^{-} at the CCSD(T)/6-311+G(d)//PBE0/6-311+G(d) level. The upper row is top view and the bottom row is side view.

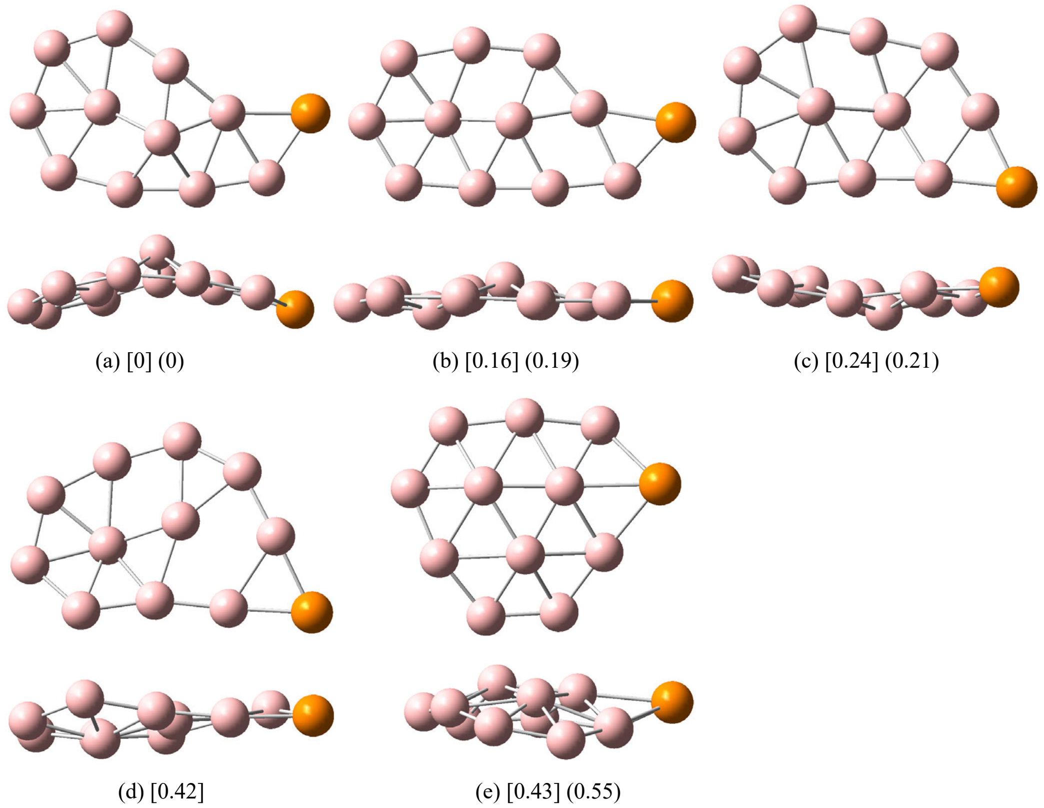

Figure S17. Low-lying isomers of doped boron clusters PB_{11}. Values in square brackets are the relative energies (eV) of the five low-energy isomers for PB_{11} at the PBE0/6-311+G(d) level. Values in parentheses are the relative energies (eV) of the five low-energy isomers for PB_{11} at the CCSD(T)/6-311+G(d)//PBE0/6-311+G(d) level. The upper row is top view and the bottom row is side view.

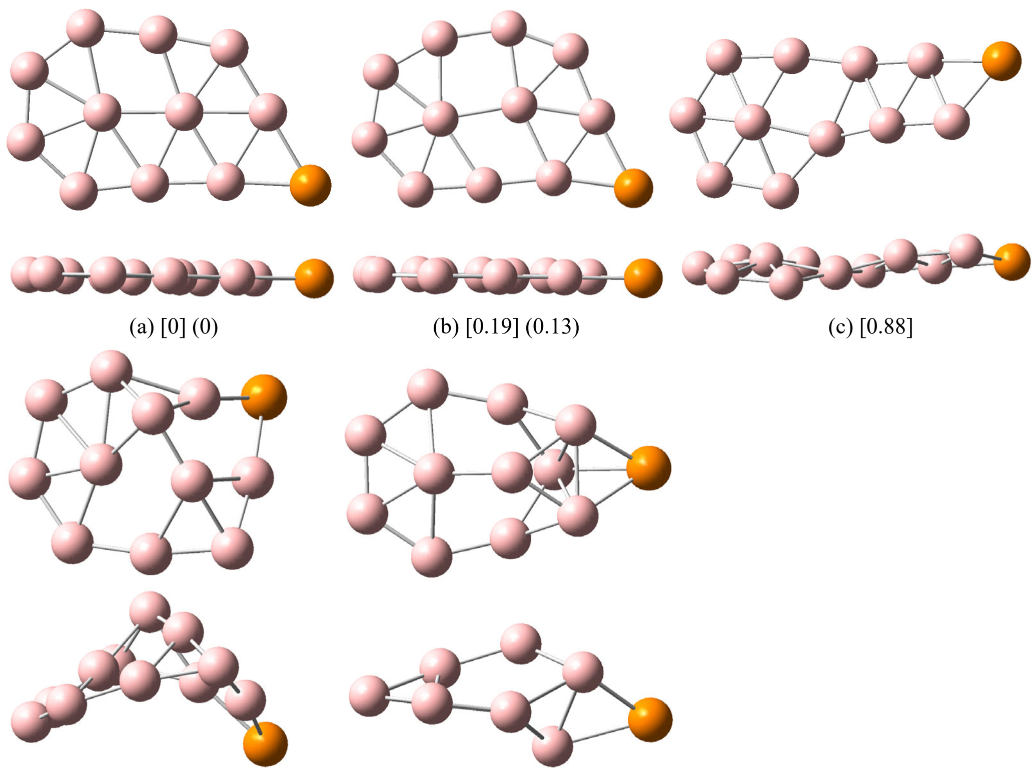

(a) [0] (0) (b) [0.19] (0.13) (c) [0.88]

(d) [1.09] (0.97) (e) [1.19] (1.02)

Figure S18. Low-lying isomers of doped boron clusters PB_{11}^{-}. Values in square brackets are the relative energies (eV) of the five low-energy isomers for PB_{11}^{-} at the PBE0/6-311+G(d) level. Values in parentheses are the relative energies (eV) of the five low-energy isomers for PB_{11}^{-} at the CCSD(T)/6-311+G(d)//PBE0/6-311+G(d) level. The upper row is top view and the bottom row is side view.

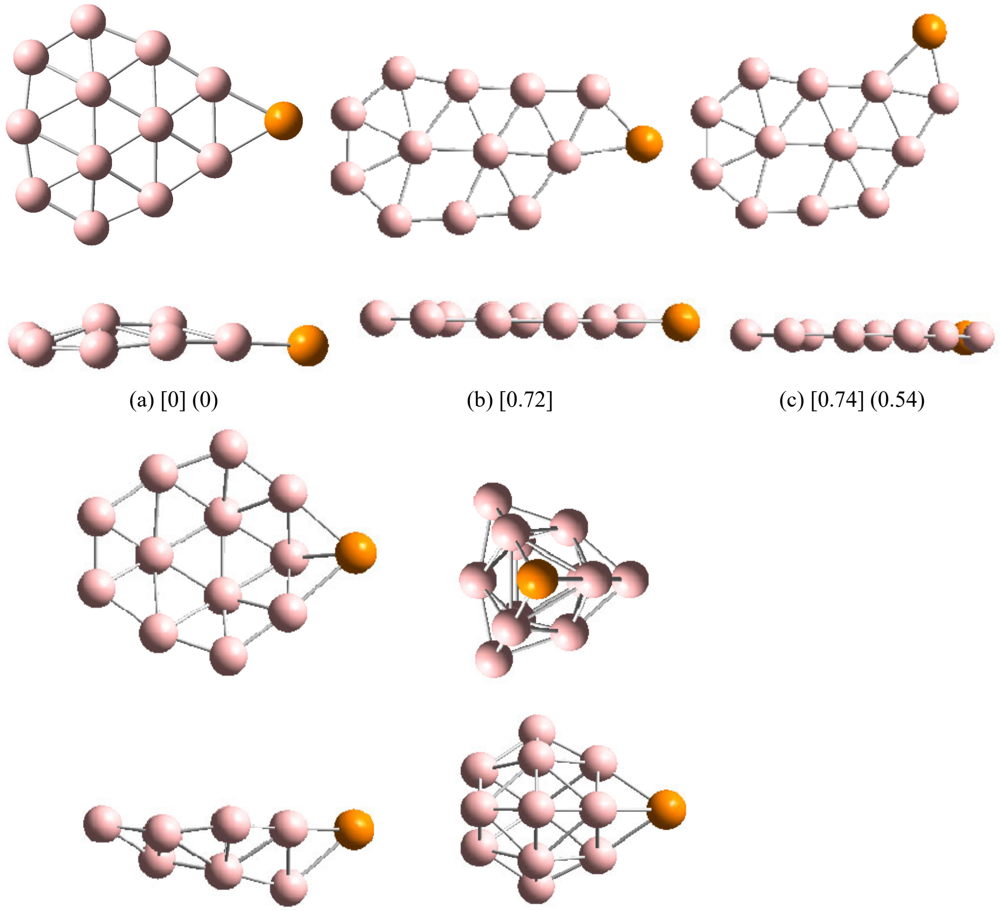

(a) [0] (0) (b) [0.72] (c) [0.74] (0.54)

(d) [1.01] (0.92) (e) [1.08] (1.22)

Figure S19. Low-lying isomers of doped boron clusters PB_{12}. Values in square brackets are the relative energies (eV) of the five low-energy isomers for PB_{12} at the PBE0/6-311+G(d) level. Values in parentheses are the relative energies (eV) of the five low-energy isomers for PB_{12} at the CCSD(T)/6-311+G(d)//PBE0/6-311+G(d) level. The upper row is top view and the bottom row is side view.

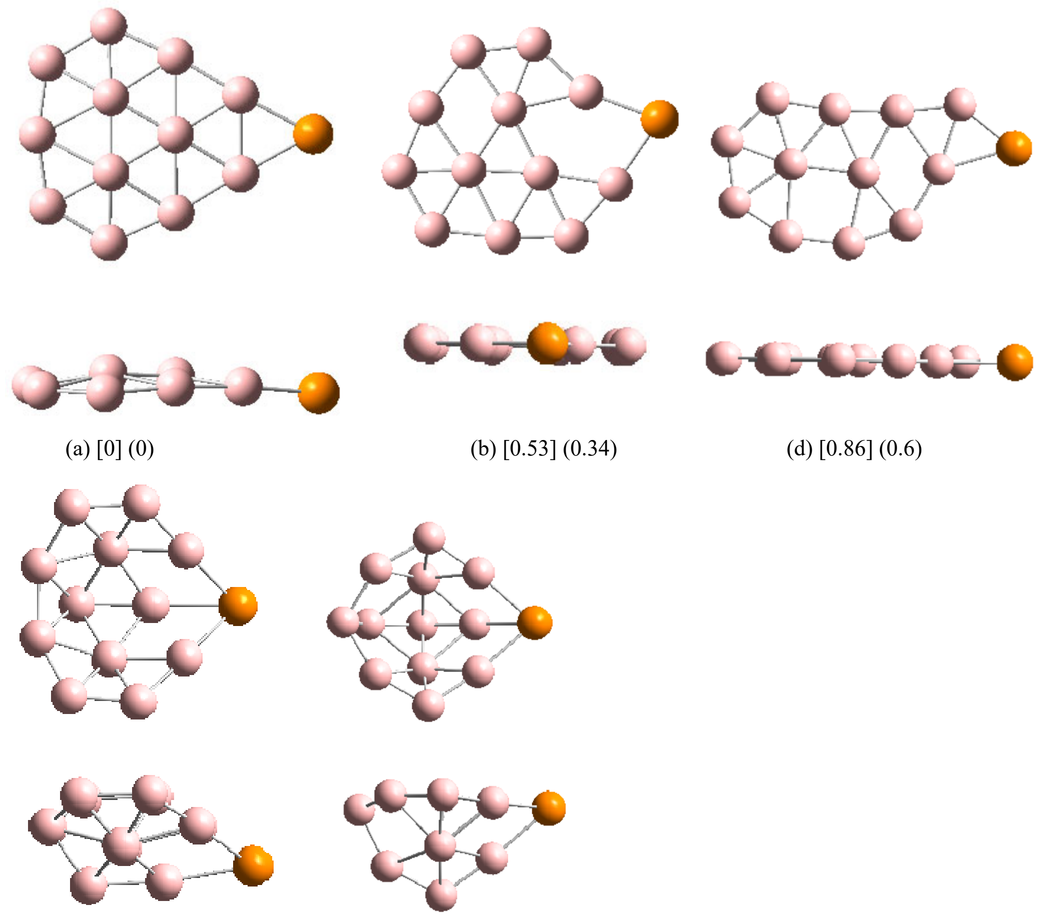

(a) [0] (0) (b) [0.53] (0.34) (d) [0.86] (0.6)

(c) [0.78] (0.73) (e) [1.04] (0.86)

Figure S20. Low-lying isomers of doped boron clusters PB_{12}^{-}. Values in square brackets are the relative energies (eV) of the five low-energy isomers for PB_{12}^{-} at the PBE0/6-311+G(d) level. Values in parentheses are the relative energies (eV) of the five low-energy isomers for PB_{12}^{-} at the CCSD(T)/6-311+G(d)//PBE0/6-311+G(d) level. The upper row is top view and the bottom row is side view.

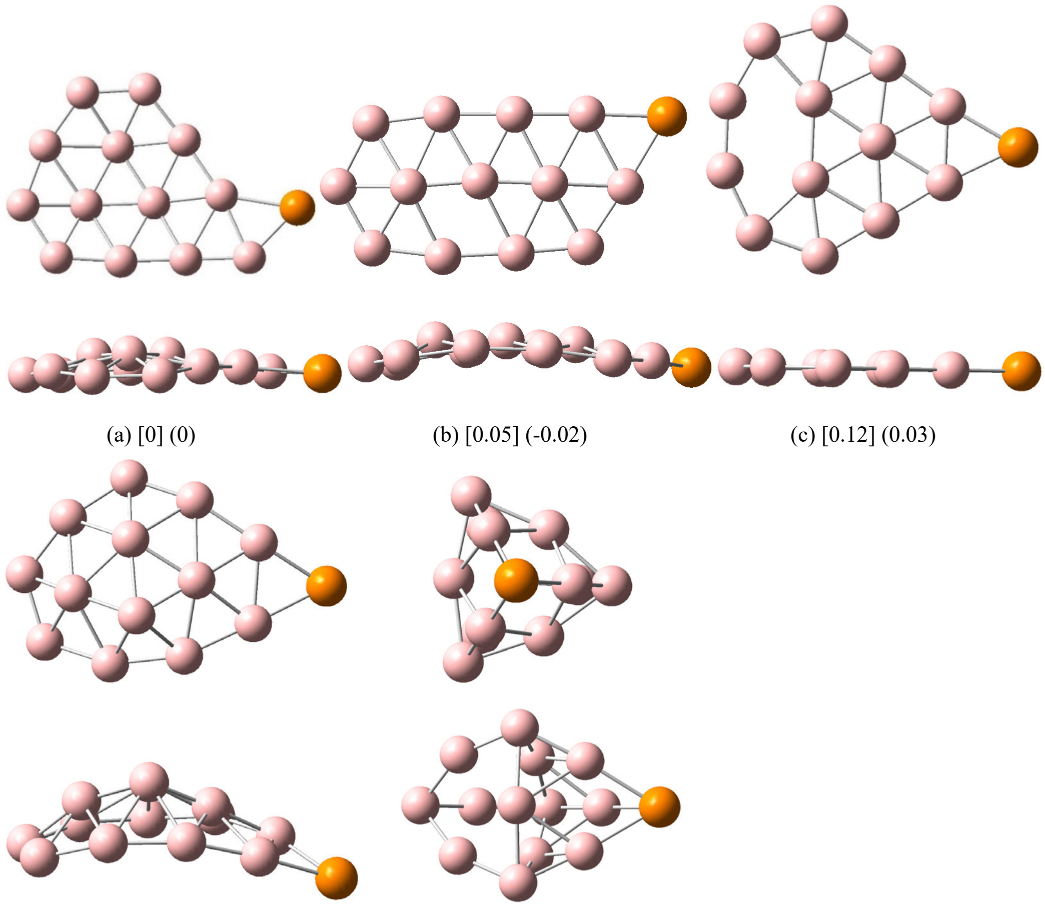

(a) [0] (0) (b) [0.05] (-0.02) (c) [0.12] (0.03)

(d) [0.21] (0.24) (e) [0.05] (0.5)

Figure S21. Low-lying isomers of doped boron clusters PB_{13}. Values in square brackets are the relative energies (eV) of the five low-energy isomers for PB_{13} at the PBE0/6-311+G(d) level. Values in parentheses are the relative energies (eV) of the five low-energy isomers for PB_{13} at the CCSD(T)/6-311+G(d)//PBE0/6-311+G(d) level. The upper row is top view and the bottom row is side view.

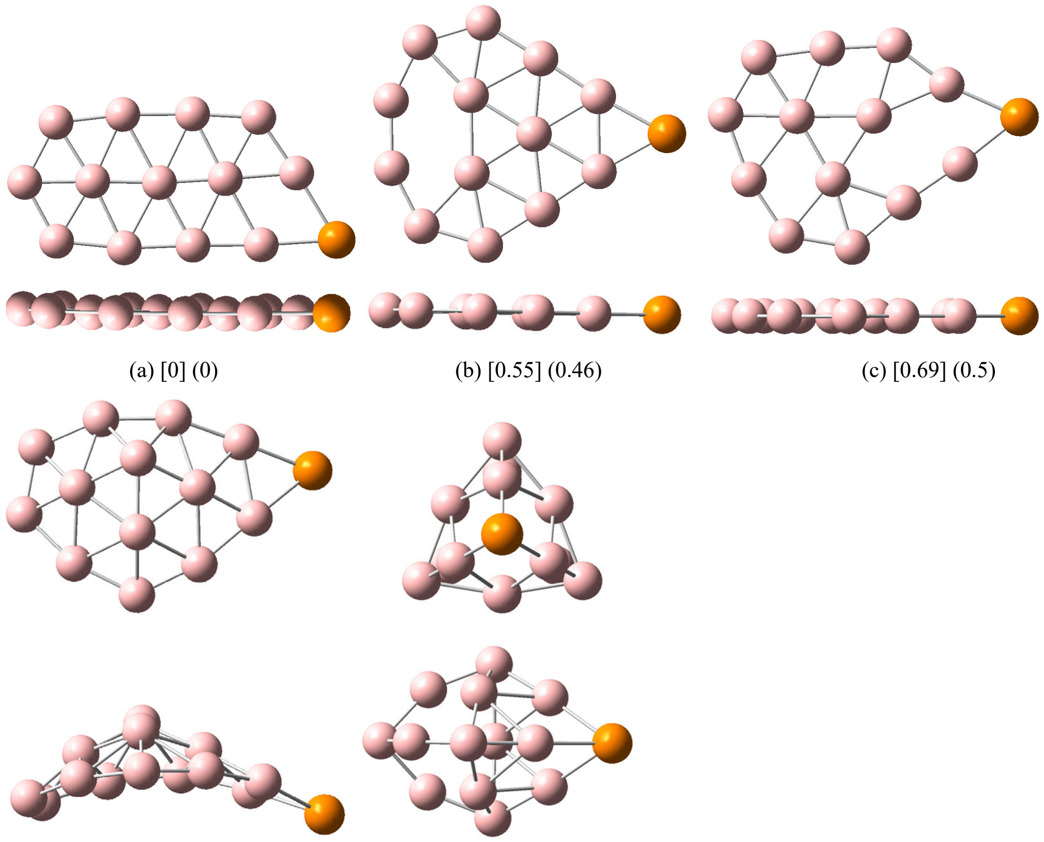

(a) [0] (0) (b) [0.55] (0.46) (c) [0.69] (0.5)

(d) [0.82] (0.86) (e) [0.96] (1.09)

Figure S22. Low-lying isomers of doped boron clusters PB_{13}^{-}. Values in square brackets are the relative energies (eV) of the five low-energy isomers for PB_{13}^{-} at the PBE0/6-311+G(d) level. Values in parentheses are the relative energies (eV) of the five low-energy isomers for PB_{13}^{-} at the CCSD(T)/6-311+G(d)//PBE0/6-311+G(d) level. The upper row is top view and the bottom row is side view.

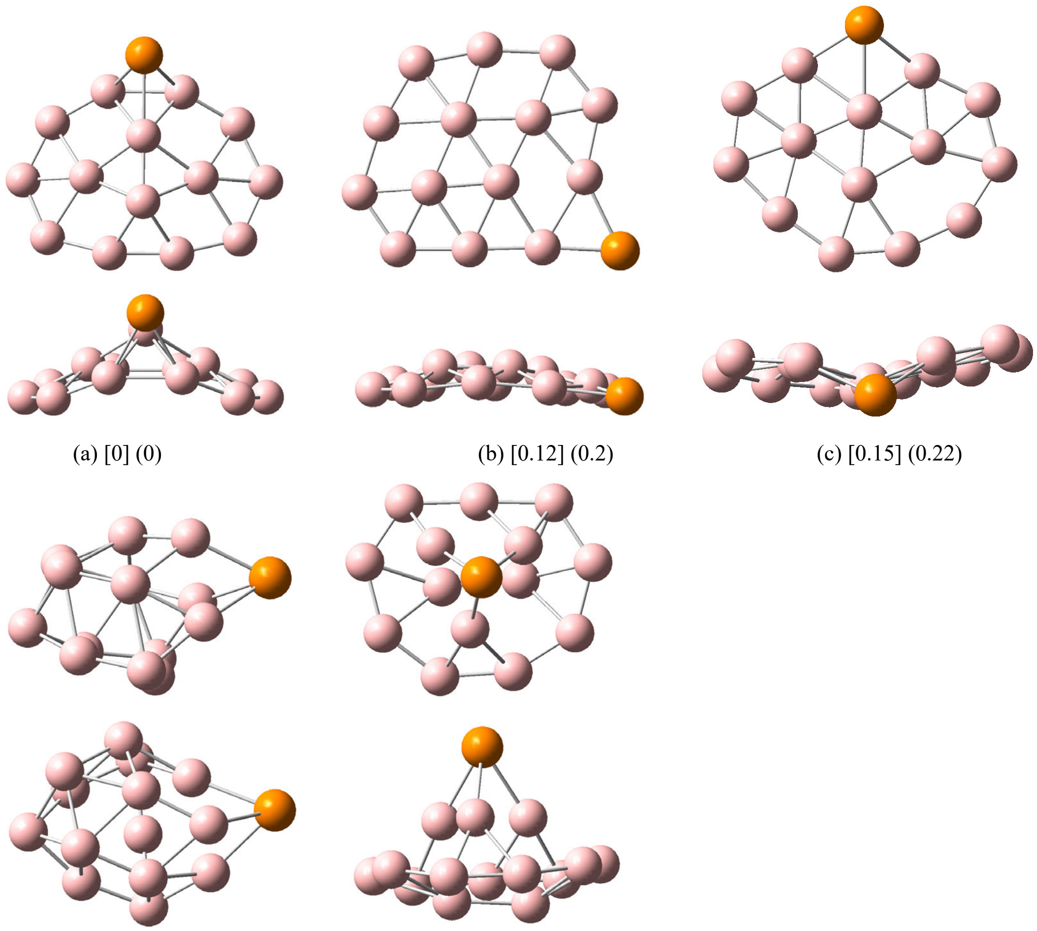

(a) [0] (0) (b) [0.12] (0.2) (c) [0.15] (0.22)

(d) [0.22] (0.39) (e) [0.34] (0.46)

Figure S23. Low-lying isomers of doped boron clusters PB_{14}. Values in square brackets are the relative energies (eV) of the five low-energy isomers for PB_{14} at the PBE0/6-311+G(d) level. Values in parentheses are the relative energies (eV) of the five low-energy isomers for PB_{14} at the CCSD(T)/6-311+G(d)//PBE0/6-311+G(d) level. The upper row is top view and the bottom row is side view.

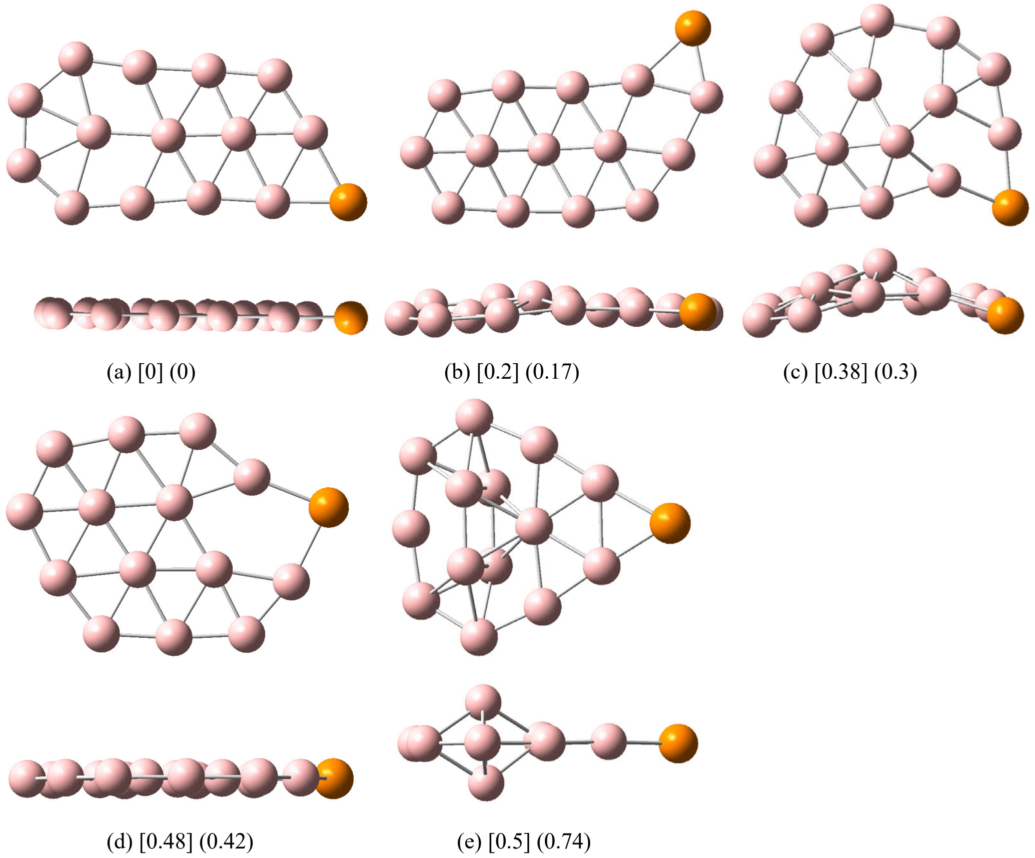

Figure S24. Low-lying isomers of doped boron clusters PB_{14}^{-}. Values in square brackets are the relative energies (eV) of the five low-energy isomers for PB_{14}^{-} at the PBE0/6-311+G(d) level. Values in parentheses are the relative energies (eV) of the five low-energy isomers for PB_{14}^{-} at the CCSD(T)/6-311+G(d)//PBE0/6-311+G(d) level. The upper row is top view and the bottom row is side view.

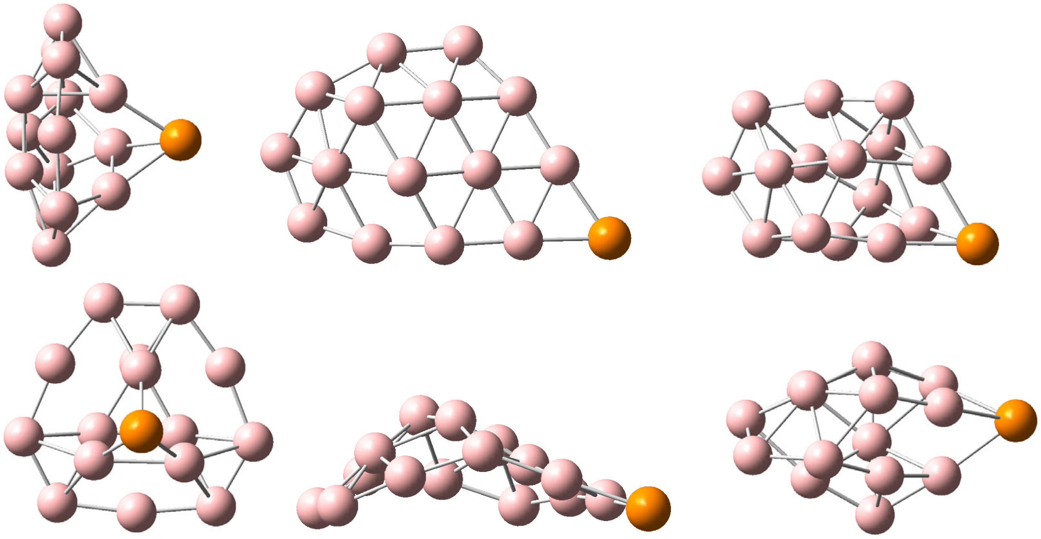

(a) [0] (0)　(b) [0.1] (0.04)　(c) [0.1] (0.12)

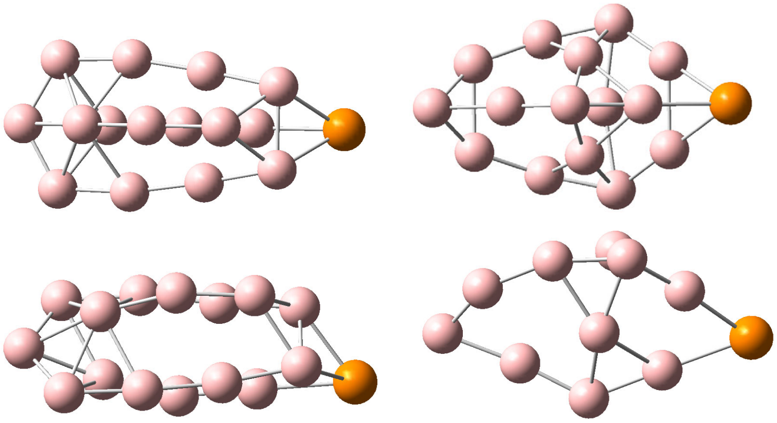

(d) [0.23] (0.15)　(e) [0.23] (0.37)

Figure S25. Low-lying isomers of doped boron clusters PB_{15}. Values in square brackets are the relative energies (eV) of the five low-energy isomers for PB_{15} at the PBE0/6-311+G(d) level. Values in parentheses are the relative energies (eV) of the five low-energy isomers for PB_{15} at the CCSD(T)/6-311+G(d)//PBE0/6-311+G(d) level. The upper row is top view and the bottom row is side view.

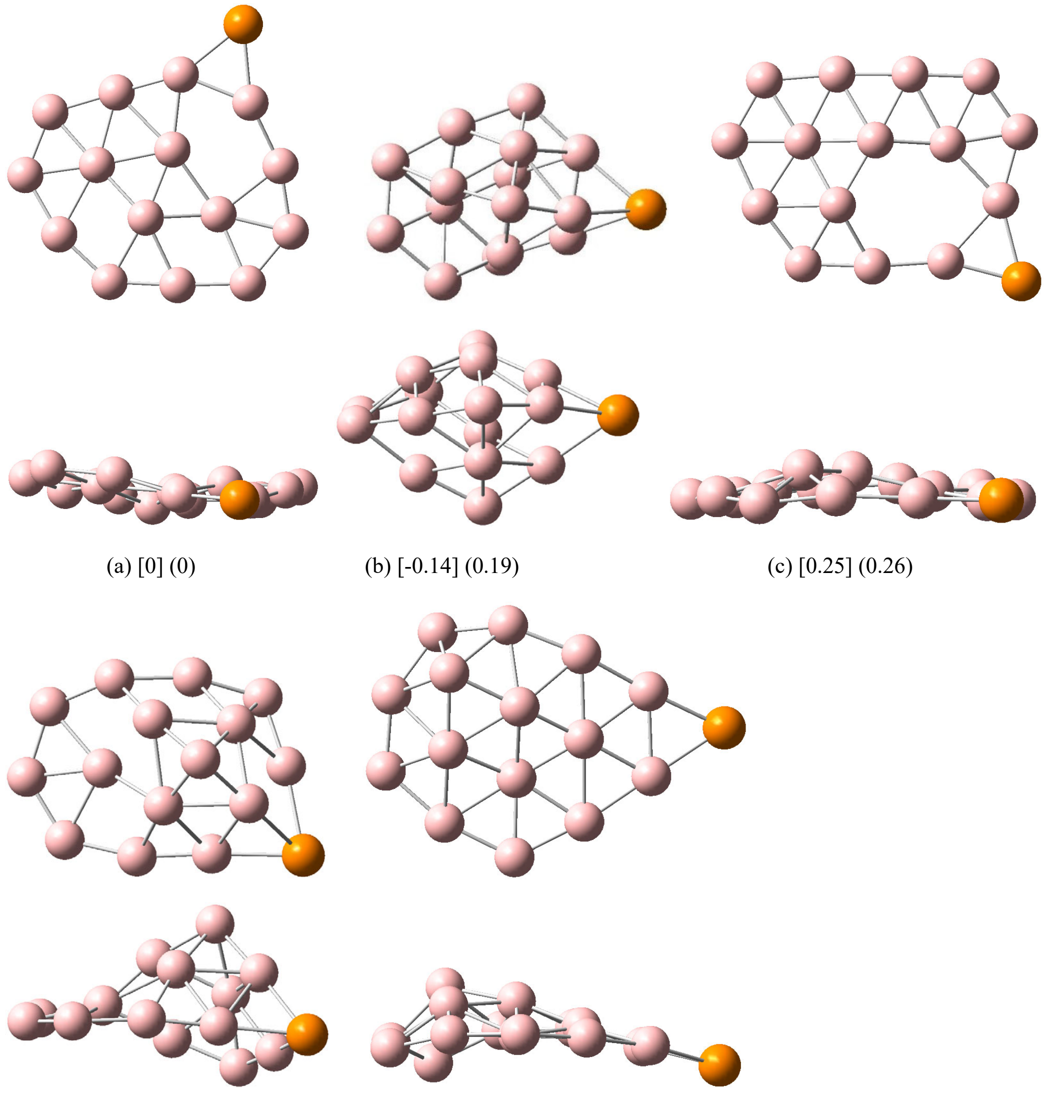

(a) [0] (0) (b) [-0.14] (0.19) (c) [0.25] (0.26)

(d) [0.15] (0.28) (e) [0.21] (0.45)

Figure S26. Low-lying isomers of doped boron clusters PB_{15}^{-}. Values in square brackets are the relative energies (eV) of the five low-energy isomers for PB_{15}^{-} at the PBE0/6-311+G(d) level. Values in parentheses are the relative energies (eV) of the five low-energy isomers for PB_{15}^{-} at the CCSD(T)/6-311+G(d)//PBE0/6-311+G(d) level. The upper row is top view and the bottom row is side view.

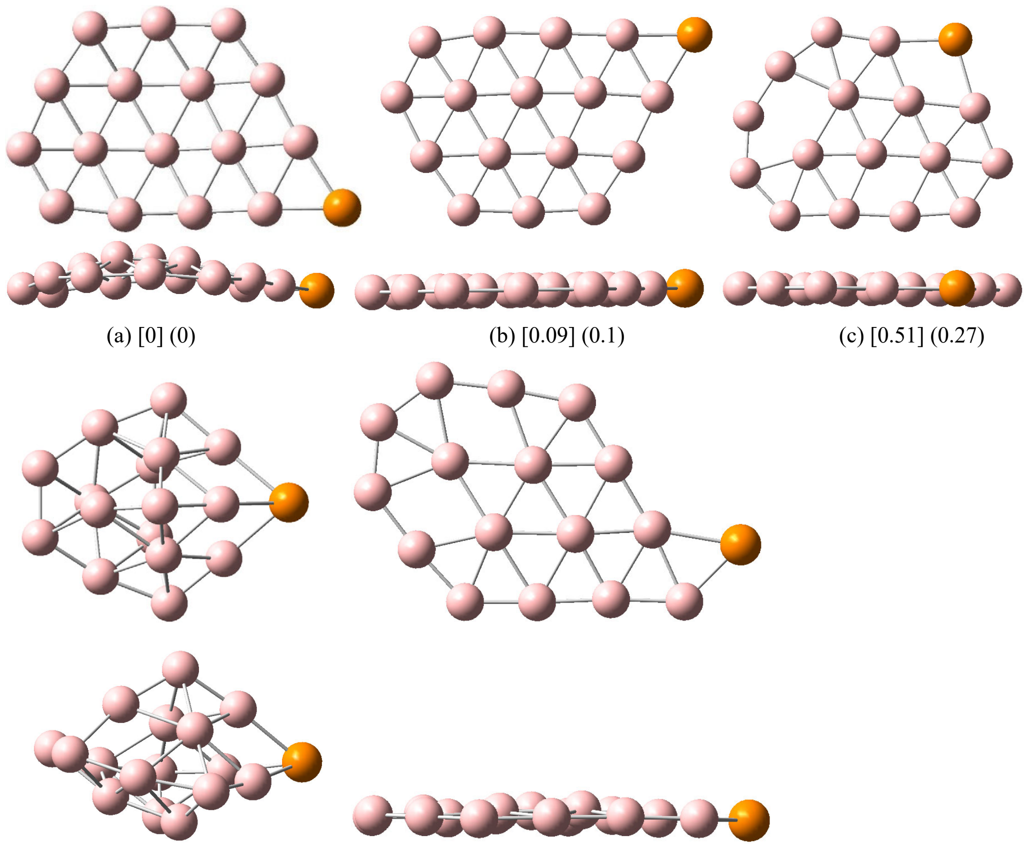

(a) [0] (0) (b) [0.09] (0.1) (c) [0.51] (0.27)

(d) [0.26] (0.32) (e) [0.61] (0.38)

Figure S27. Low-lying isomers of doped boron clusters PB_{16}. Values in square brackets are the relative energies (eV) of the five low-energy isomers for PB_{16} at the PBE0/6-311+G(d) level. Values in parentheses are the relative energies (eV) of the five low-energy isomers for PB_{16} at the CCSD(T)/6-311+G(d)//PBE0/6-311+G(d) level. The upper row is top view and the bottom row is side view.

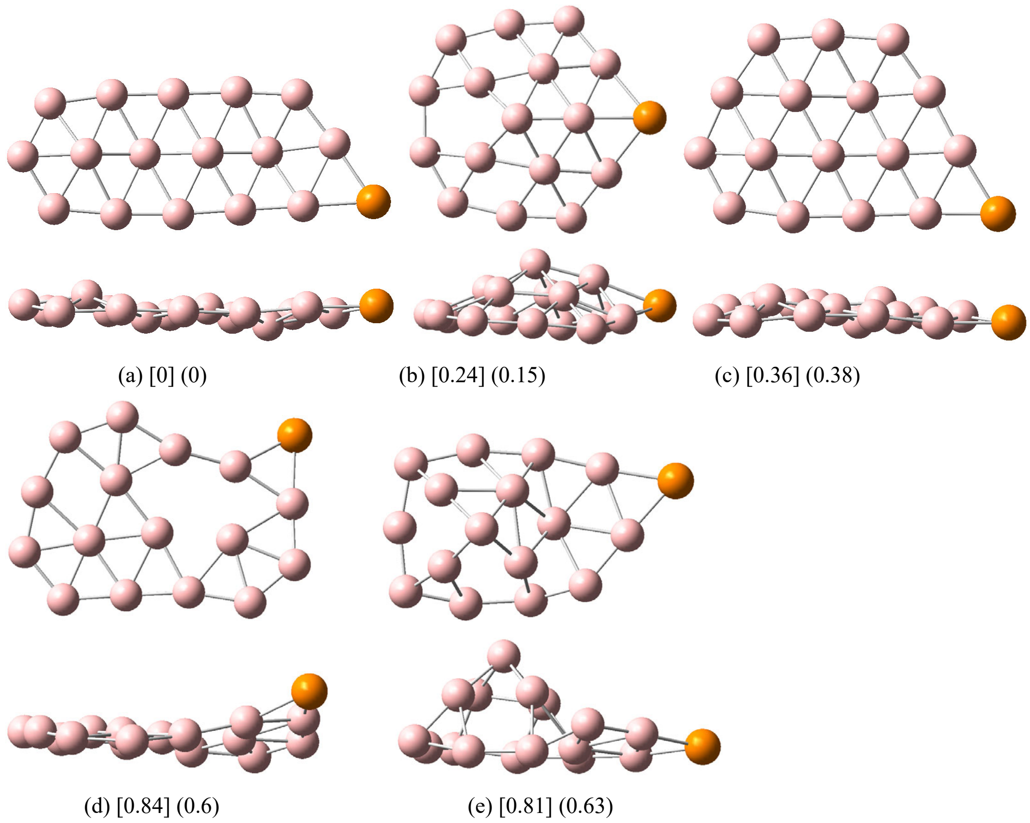

Figure S28. Low-lying isomers of doped boron clusters PB_{16}^{-}. Values in square brackets are the relative energies (eV) of the five low-energy isomers for PB_{16}^{-} at the PBE0/6-311+G(d) level. Values in parentheses are the relative energies (eV) of the five low-energy isomers for PB_{16}^{-} at the CCSD(T)/6-311+G(d)//PBE0/6-311+G(d) level. The upper row is top view and the bottom row is side view.

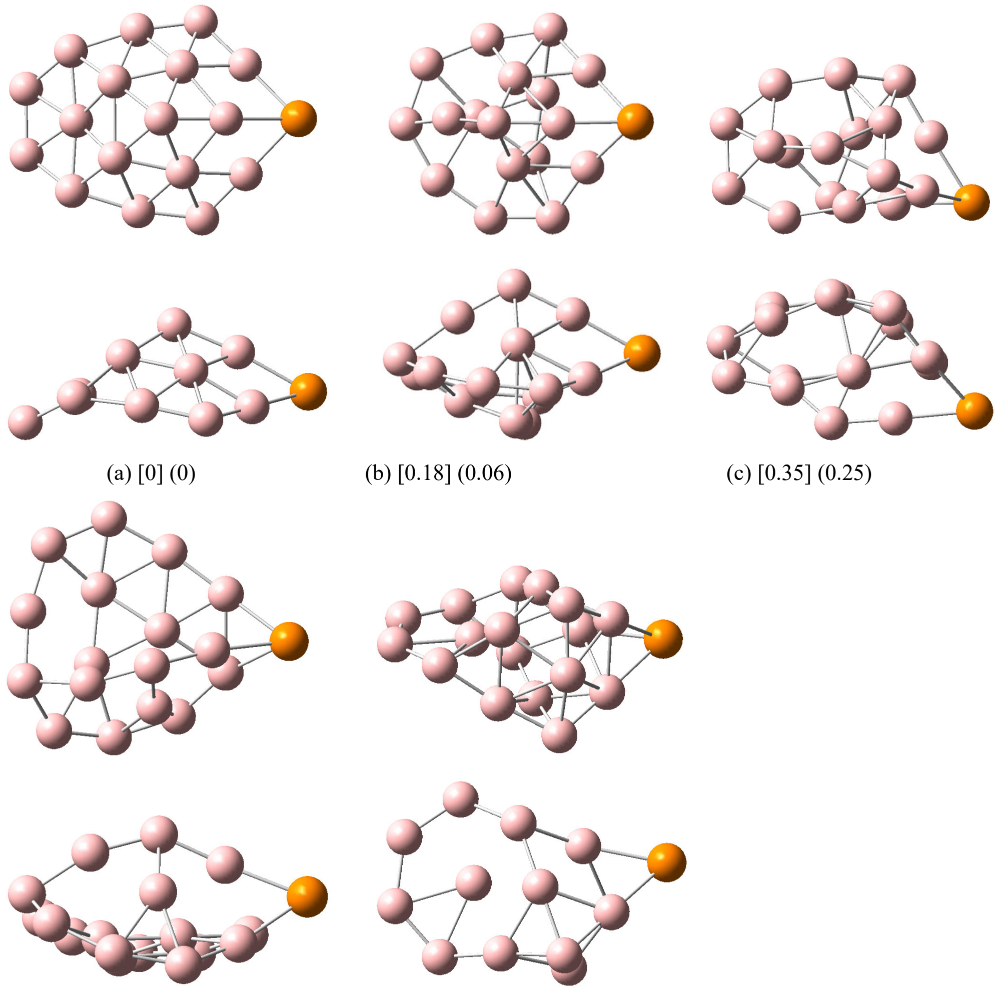

(a) [0] (0) (b) [0.18] (0.06) (c) [0.35] (0.25)

(d) [0.56] (0.35) (e) [0.45] (0.46)

Figure S29. Low-lying isomers of doped boron clusters PB_{17}. Values in square brackets are the relative energies (eV) of the five low-energy isomers for PB_{17} at the PBE0/6-311+G(d) level. Values in parentheses are the relative energies (eV) of the five low-energy isomers for PB_{17} at the CCSD(T)/6-311+G(d)//PBE0/6-311+G(d) level. The upper row is top view and the bottom row is side view.

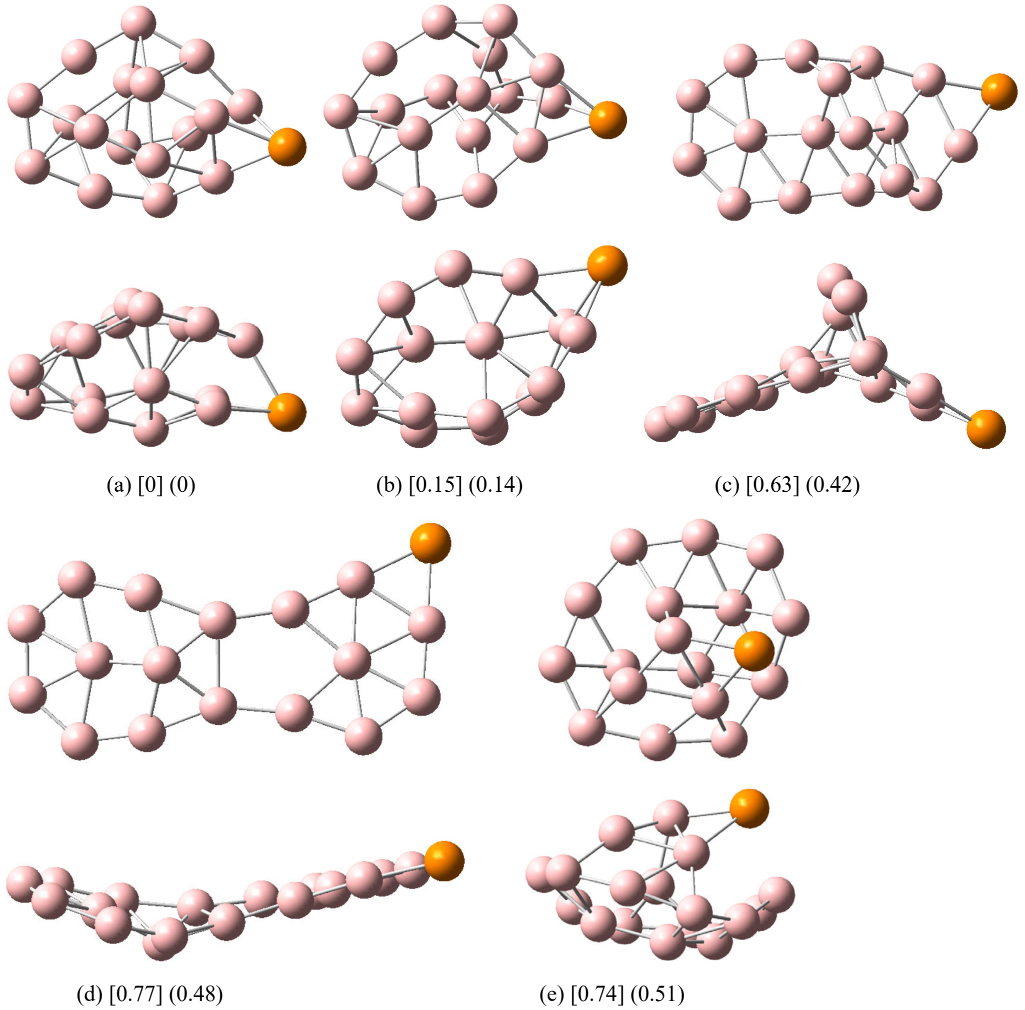

Figure S30. Low-lying isomers of doped boron clusters PB_{17}^{-}. Values in square brackets are the relative energies (eV) of the five low-energy isomers for PB_{17}^{-} at the PBE0/6-311+G(d) level. Values in parentheses are the relative energies (eV) of the five low-energy isomers for PB_{17}^{-} at the CCSD(T)/6-311+G(d)//PBE0/6-311+G(d) level. The upper row is top view and the bottom row is side view.

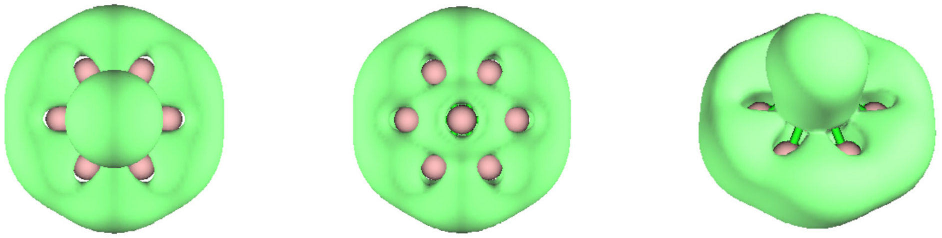

(top) (bottom) (side)

Figure S31. Electron localization function (ELF) of PB_7 with the isovalue set to 0.59.

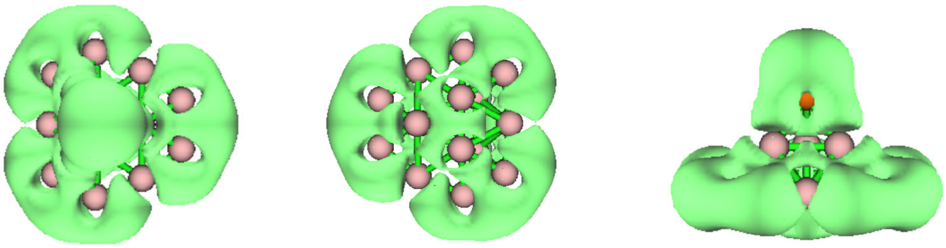

(top) (bottom) (side)

Figure S32. Electron localization function (ELF) of PB_{15} with the isovalue set to 0.7.

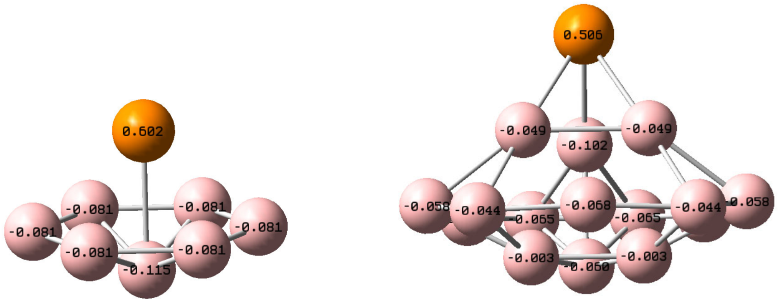

Figure S33 Charge population of PB_7 and PB_{15}

Table S1 The First VDE values of bare B clusters and P-doped boron clusters

	The First VDE (eV)		The First VDE (eV)
B_3^-	2.82	PB_3^-	2.19
B_4^-	1.99	PB_4^-	1.78
B_5^--	2.4	PB_5^--	3.33
B_6^-	3.01	PB_6^-	2.47
B_7^-	2.85	PB_7^-	3.43
B_8^-	3.02	PB_8^-	3.24
B_9^-	3.46	PB_9^-	3.55
B_{10}^-	3.06	PB_{10}^-	3.35
B_{11}^-	3.426	PB_{11}^-	3.47
B_{12}^-	2.26	PB_{12}^-	3.59
B_{13}^-	3.78	PB_{13}^-	3.44
B_{14}^-	3.102	PB_{14}^-	3.51
B_{15}^-	3.43	PB_{15}^-	3.53
B_{16}^-	3.39	PB_{16}^-	3.88
